# Structure, assembly and inhibition of the *Toxoplasma gondii* respiratory chain supercomplex

**DOI:** 10.1038/s41594-025-01531-7

**Published:** 2025-05-19

**Authors:** Andrew E. MacLean, Shikha Shikha, Mariana Ferreira Silva, Max J. Gramelspacher, Aaron Nilsen, Katherine M. Liebman, Sovitj Pou, Rolf W. Winter, Amit Meir, Michael K. Riscoe, J. Stone Doggett, Lilach Sheiner, Alexander Mühleip

**Affiliations:** 1 School of Infection and Immunity, University of Glasgow, Glasgow, UK; 2 Glasgow Centre for Parasitology, University of Glasgow, Glasgow, UK; 3 VA Portland Health Care System, Portland, OR USA; 4 Medicinal Chemistry Core, Oregon Health and Science University, Portland, OR USA; 5 Centre for Virus Research, University of Glasgow, Glasgow, UK; 6 Department of Microbiology and Molecular Immunology, Oregon Health and Science University, Portland, OR USA; 7 School of Medicine Division of Infectious Diseases, Oregon Health and Science University, Portland, OR USA; 8 Institute of Biotechnology, Helsinki Institute of Life Science HiLIFE, University of Helsinki, Helsinki, Finland

**Keywords:** Mitochondrial proteins, Drug discovery, Cryoelectron tomography

## Abstract

The apicomplexan mitochondrial electron transport chain is essential for parasite survival and displays a divergent subunit composition. Here we report cryo-electron microscopy structures of an apicomplexan III_2_–IV supercomplex and of the drug target complex III_2_. The supercomplex structure reveals how clade-specific subunits form an apicomplexan-conserved III_2_–IV interface with a unique, kinked architecture, suggesting that supercomplexes evolved independently in different eukaryotic lineages. A knockout resulting in supercomplex disassembly challenges the proposed role of III_2_–IV in electron transfer efficiency as suggested for mammals. Nevertheless, knockout analysis indicates that III_2_–IV is critical for parasite fitness. The complexes from the model parasite *Toxoplasma gondii* were inhibited with the antimalarial atovaquone, revealing interactions underpinning species specificity. They were also inhibited with endochin-like quinolone (ELQ)-300, an inhibitor in late-stage preclinical development. Notably, in the apicomplexan binding site, ELQ-300 is flipped compared with related compounds in the mammalian enzyme. On the basis of the binding modes and parasite-specific interactions discovered, we designed more potent ELQs with subnanomolar activity against *T.* *gondii*. Our findings reveal critical evolutionary differences in the role of supercomplexes in mitochondrial biology and provide insight into cytochrome *b* inhibition, informing future drug discovery.

## Abstract (web summary)

Here the authors demonstrate that the assembly of mitochondrial respiratory supercomplex (III_2_–IV) from *Toxoplasma gondii* is critical for parasite fitness. They reveal the basis for cytochrome *b* inhibition by atovaquone and improved ELQ inhibitors.

## Main

The mitochondrial electron transport chain (mETC) is essential for nearly all organisms from the divergent domain of eukaryotes, yet our knowledge of how it works is primarily informed by studies in yeast and mammals. The occurrence of respiratory supercomplexes has been known for decades^1^ and recently, insight from structural studies exposed the interactions that mediate their formation. However, whether supercomplex formation is functionally relevant for electron transfer or whether this reoccurring arrangement confers a different advantage, such as increased complex stability, is still a subject of ongoing investigations^2–6^. This study provides an evolutionary perspective on the currently proposed supercomplex functions through the structural and functional characterization of a protozoan parasite supercomplex. Apicomplexans are parasites that belong to the myzozoan clade of eukaryotes. These parasites were recently shown to have expanded mETC complexes with numerous clade-specific subunits^7,8^; however, their role remains unknown. Likewise, in the apicomplexan mETC, complex III_2_ (CIII) is the primary target for antiparasitic drugs. However, the molecular basis for this sensitivity is not fully understood owing to the lack of a parasite CIII structure. We address both of these questions through structural analyses and genetic studies.

## Supercomplex architecture and role of supernumerary subunits

**Fig. 1:**
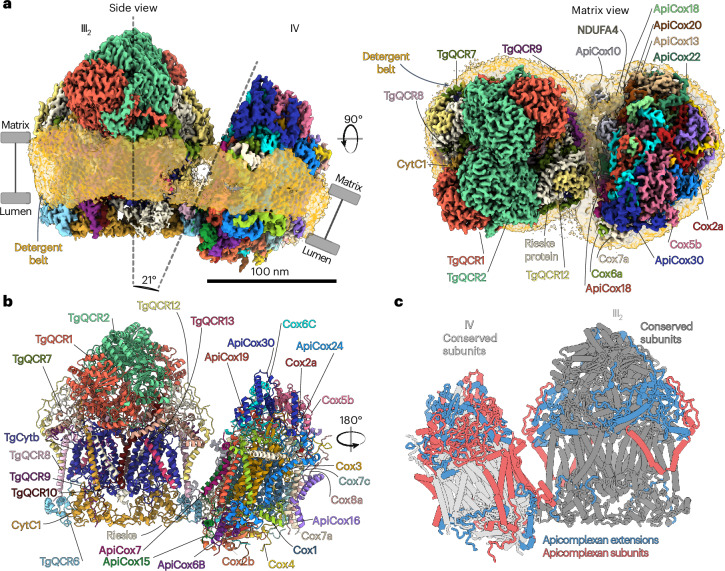
Structure of the *T.* *gondii* respiratory supercomplex. **a**, The side view (left) and top view (right) of the III_2_–IV composite map containing 13 clade-specific subunits. The transmembrane region of CIV is kinked by 21° with respect to CIII. **b**, An atomic model of III_2_–IV. **c**, Conserved and clade-specific structural elements.

**Extended Data Fig. 1:**
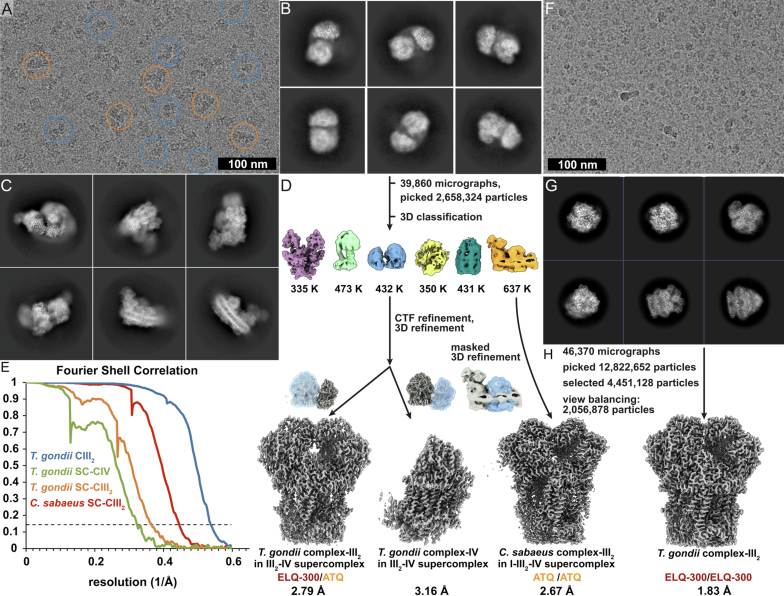
Cryo-EM structure determination workflow. **(a)** Representative micrograph with *T. gondii* and *C. sabaeus* supercomplex particles indicated in blue and orange, respectively. **(b)** Representative 2D classes of the *T. gondii* supercomplex. **(c)** Representative 2D classes of *C. sabaeus* supercomplex. **(d)** Data processing scheme, highlighting the reference-based 3D classification to separate *T. gondii* and *C. sabaeus* ATP synthase (purple, light green), mitochondrial large subunit (yellow) and putative mitochondrial chaperonin (teal) from *T. gondii* and *C. sabaeus* supercomplexes (blue and orange). **(e)** Fourier Shell Correlation (FSC) curves. **(f)** Representative micrograph with *T. gondii* complex III from IP. **(g)** Representative 2D classes of the *T. gondii* complex III. (**h**) Data processing scheme for complex III.

**Extended Data Fig. 2:**
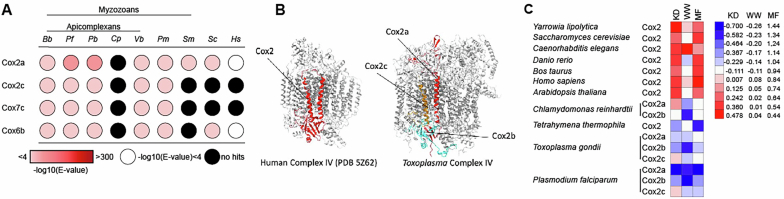
Sequence conservation of newly assigned myzozoan-specific CIV subunits and Cox2 split. **(a)** Summary of homology search outcomes for the new subunits. **(b)** Comparison of Cox2 structure in human CIV and in *Toxoplasma* where Cox2 is split in three. **(c)** Heat map indicating the mean hydrophobicity (calculated by grand average of hydropathy (KD)^65^ or according to Wimley-White (WW)^66^ or the Moon-Fleming (MF)^67^ of Cox2 homologs in divergent organisms. Source data

The *Toxoplasma gondii* respiratory supercomplex was purified from the rapidly proliferating tachyzoite stage. Using single-particle cryo-electron microscopy (cryo-EM), we determined the structure of the III_2_–IV supercomplex in the presence of atovaquone and endochin-like quinolone (ELQ)-300 (10 µM each) at 2.8 Å resolution, allowing atomic model construction (Fig. 1a,b, Table 1 and Extended Data Fig. 1). The 960 kDa supercomplex consists of 37 subunits and contains the conserved reaction centers and cofactors (Supplementary Table 1). In total, 13 subunits are apicomplexan conserved, of which 2 are found in CIII (TgQCR12 and 13). Unlike previously predicted^8,9^, the complex contains a newly assigned homolog of QCR10, which could only be identified via structural similarity. Complex IV (CIV) is greatly augmented, containing 11 apicomplexan-conserved subunits and substantial clade-specific subunit extensions (Fig. 1c and Supplementary Table 1), giving it a molecular mass of 405 kDa, much larger than the 207 kDa mammalian homolog^10^. For the 11 apicomplexan-conserved subunits of *T.* *gondii* CIV (ApiCox7, 10, 13, 15, 16, 18, 19, 20, 22, 24 and 30), we extend an existing nomenclature indicating the approximate molecular weight^11^ (Methods and Supplementary Table 1). The increased molecular masses of CIII and CIV are in line with the larger complex II and adenosine triphosphate (ATP) synthase assemblies found in Apicomplexa^12,13^. These findings confirm that the apicomplexan oxidative phosphorylation complexes display augmented subunit compositions, raising questions about the role of new subunits and extensions, as well as their evolution. An overall reduced protein hydrophobicity resulting from a splitting of structural elements into several subunits may enable mitochondrial protein targeting following gene transfer from the mitochondrial genome to the nuclear genome^13,14^. In our structure, *T.* *gondii* Cox2 is split into three proteins, each providing canonical helices but displaying reduced overall hydrophobicity compared with canonical Cox2 (Extended Data Fig. 2), providing support that this mechanism enabled the marked mitochondrial genome reduction in apicomplexans to just three protein-coding genes.

**Table 1:** Cryo-EM data collection, refinement and validation statistics

	*T. gondii* CIII_2_ (EMDB 52621), (PDB 9I4X)	*T. gondii* CIV (EMDB 52622), (PDB 9I4Y)	*C. sabaeus* CIII_2_ (EMDB 51939), (PDB 9H8T)	*T. gondii* CIII_2_ ELQ-300 (EMDB 51157), (PDB 9G9T)
**Data collection and processing**				
Magnification	165,000	165,000	165,000	165,000
Voltage (kV)	300	300	300	300
Electron exposure (e^−^ Å^−2^)	36	36	36	36
Defocus range (μm)	0.6–1.8	0.6–1.8	0.6–1.8	0.6–1.6
Pixel size (Å)	0.83	0.83	0.83	0.3
Symmetry imposed	C2	C1	C1	C2
Initial particle images (no.)	2,658,324	2,658,324	2,658,324	4,451,128
Final particle images (no.)	432,158	432,158	637,287	2,056,878
Map resolution (Å)	2.79	3.16	2.67	1.83
FSC threshold	0.143	0.143	0.134	0.143
Map resolution range (Å)	2.6–3.2	2.45–8.18	2.6–2.93	1.74–2.06
**Refinement**				
Initial model used (PDB code)	AlphaFold + de novo	AlphaFold + de novo	7o37	AlphaFold + de novo
Model resolution (Å)	3.6	3.1	2.83	1.9
FSC threshold	0.5	0.5	0.5	0.5
Model resolution range (Å)	2.6–3.2	2.45–8.18	2.6–2.93	1.74–2.06
Map sharpening *B* factor (Å^2^)	78.7	70.4	99.5	45.6
Model composition				
Non-hydrogen atoms	83,424	60,600	67,256	82.473
Protein residues	5,060	3,515	4,091	4,830
Ligands	18	22	12	18
*B* factors (Å^2^)				
Protein	39.40	57.54	32.53	8.94
Ligand	33.65	59.96	28.56	14.20
Root mean square deviations				
Bond lengths (Å)	0.003	0.014	0.002	0.003
Bond angles (°)	0.493	1.157	0.524	0.555
Validation				
MolProbity score	1.44	1.29	1.12	1.18
Clashscore	5.18	2.34	2.02	1.9
Poor rotamers (%)	1.01	0.65	0.63	0.43
Ramachandran plot				
Favored (%)	97.12	96.02	97.21	96.48
Allowed (%)	2.84	3.61	2.74	3.31
Disallowed (%)	0.04	0.38	0.05	0.21

**Fig. 2:**
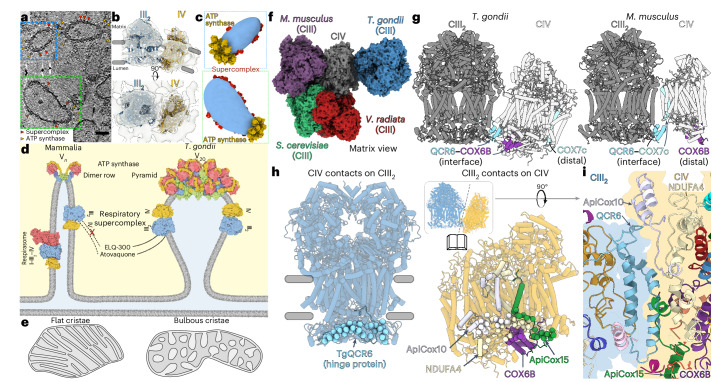
The lumenal III_2_–IV interface generates an apicomplexan-specific supercomplex architecture that reflects cristae morphology. **a**, A slice of an electron cryo-tomogram of mitochondrial membranes from *T.* *gondii*. ATP synthase (yellow) and supercomplexes (red) are indicated. **b**, The subtomogram average map (gray) is consistent with a III_2_–IV_1_ arrangement. The individually fitted CIII dimer (blue) and CIV monomer structures (yellow) are from *S. cerevisiae* (PDB 6T15). **c**, A three-dimensional close-up of **a** showing apical ATP synthase and lateral supercomplex. The apices of the mitochondrial membranes (blue) are occupied by ATP synthase pyramids (yellow, described in ref. ^13^), with supercomplexes (red) in the flatter lateral regions. Both structures were obtained by subtomogram averaging. **d**, The arrangement of ATP synthase and supercomplexes in cristae of mammals and *T.* *gondii*. The rim of flat cristae in mammals are in line with ATP synthase rows (PDB 7ajb), whereas the apices of bulbous *T.* *gondii* cristae are shaped by ATP synthase pyramids (PDB 6TML). Mammalian supercomplexes (PDB 7o37) are found in the lateral, flat membrane regions, whereas the kinked III_2_–IV *T.* *gondii* supercomplex (this study) is accommodated by the curved, lateral regions. The matrix is shown in yellow. The cristae lumen and intermembrane space are shown in blue. **e**, A schematic highlighting the resulting cristae morphologies, adapted from ref. ^43^. **f**, Overlay of supercomplex structures aligned on CIV highlights different architectures, as revealed by differing positions of CIII from mammals (*Mus musculus*, PDB 7o37 mature supercomplex), yeast (*S.* *cerevisiae*, PDB 6giq) and plant (*Vigna radiata*, PDB 7jrp). The mammalian and *T.* *gondii* CIII homologs bind to opposite sides of CIV. **g**, Comparison of the *T.* *gondii* (this study) and *M.* *musculus* III_2_–IV (PDB 7o37) showing the rotation of CIV relative to CIII, thereby placing different CIV subunits (COX6B and COX7c) at the interface with QCR6, or at the distal end of the supercomplex. **h**, Open-book view of the III_2_–IV interface with lumenal contacts with respective subunits highlighted by spheres. **i**, Top view of the III_2_–IV interface.

**Extended Data Fig. 3:**
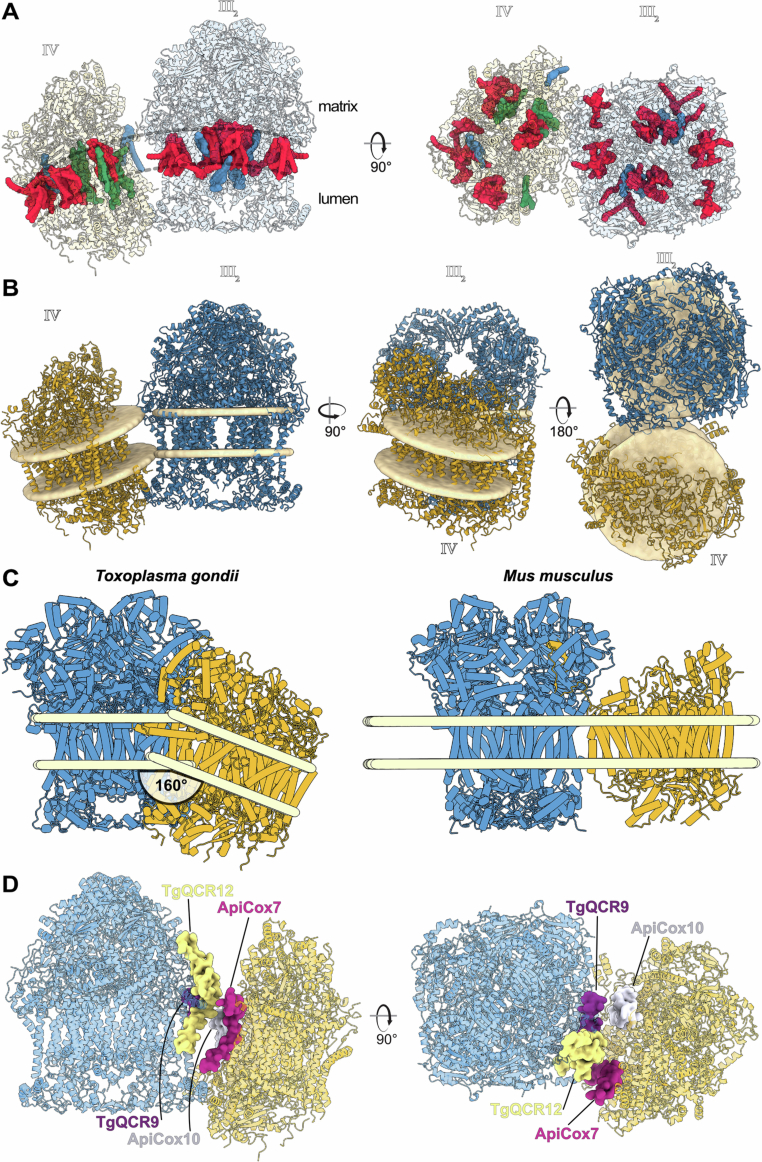
Lipids and kinked architecture of the supercomplex. **(a)** Bound lipids of the III_2_-IV supercomplex with cardiolipins shown in red, phosphatidylcholine in blue and phosphatidylethanolamine in green. Lipid positions indicate a bend membrane region (dashed lines). **(b)** *T. gondii* supercomplex structure with estimated membrane plane regions showing a rotational offset between the two subcomplexes CIII and CIV. **(c)** Comparison of the kinked *T. gondii* supercomplex architecture the flat assembly of the murine III_2_-IV supercomplex (PDB 7O3C). **(d)** Steric hindrance between TgQCR12 and ApiCox7 and between ApiCox10 and TgQCR9 prevents a flat assembly of the III_2_-IV supercomplex.

Supernumerary subunits may also mediate higher-order oxidative phosphorylation assemblies that suit clade-specific mitochondrial functions and contribute to membrane curvature induction. Our *T.* *gondii* III_2_–IV structure reveals an unusual supercomplex architecture, in which the transmembrane region of CIV is tilted against the membrane plane of CIII by ~20° (Fig. 1a and Extended Data Fig. 3). This finding is further supported by the positions of lipid-binding sites within the curved membrane region (Extended Data Fig. 3a,b). By contrast, the transmembrane region of the mammalian and yeast supercomplex homologs are flat (Extended Data Fig. 3c). The kink is probably induced by the presence of clade-specific subunits TgQCR12 in CIII and ApiCox7 in CIV, which act as spacers in the transmembrane and matrix regions and would clash in a flat architecture, as present in the mammalian supercomplex (Extended Data Fig. 3d). Recently, kinked interfaces have been observed between complexes I and III_2_ in ciliate and plant supercomplexes^15,16^ and the ciliate I–II–III_2_–IV_2_ structure was shown to contribute to membrane curvature induction. In *T.* *gondii*, pentagonal pyramid arrays of ATP synthase generate a distinct bulbous cristae morphology that is characteristic for mitochondria of apicomplexan parasites^13^. Using electron cryo-tomography and subtomogram averaging, we identified the III_2_–IV supercomplex in situ and revealed its localization in the curved, lateral regions of the cristae membranes (Fig. 2a–c). Therefore, the newly observed kinked III_2_–IV architecture, mediated by clade-specific subunits, probably reflects the curvature of the lateral cristae regions. This architecture differs markedly from the lamellar cristae found in mammals, where respiratory supercomplexes reside in the flat membrane regions^17^ (Fig. 2d,e).

## A clade-specific interface is critical for parasite fitness

**Extended Data Fig. 4:**
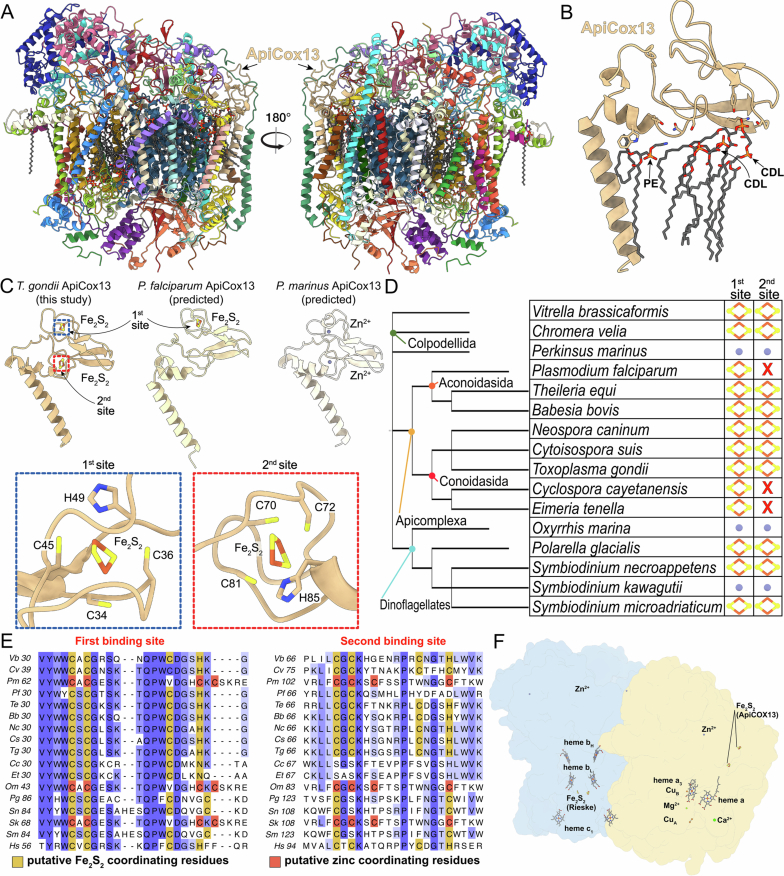
Conservation and metal ligand binding of myozoan Cox13. **(a)** *T. gondii* CIV structure **(b)** *Tg*ApiCox13 with bound lipids (CDL, cardiolipin; PE, phosphatidylethanolamine) **(c)** ApiCox13 from *T. gondii* (left, this study), predicted homolog structures from *Perkinsus marinus* and *Plasmodium falciparum*. Of the two Fe_2_S_2_ clusters in *T. gondii*, one is conserved in *P. falciparum*, whereas *P. marinus* seem to utilize zinc. Insets show Fe_2_S_2_ coordination sites. **(d)** Phylogenetic tree with conservation of Fe_2_S_2_ and zinc **(e)** Multi-sequence alignment with residues involved in Fe_2_S_2_ or zinc binding shown in yellow and orange respectively. **(f)** Cofacors of the *T. gondii* respiratory supercomplex. The Zn^2+^ of the MPP-beta subunits and the Fe_2_S_2_ iron-sulfur clusters in *Tg*ApiCox13 that are not part of the electron transfer pathway are indicated with respective subunit names. Source data

The *T.* *gondii* CIV is unusual in its augmented subunit composition and orientation within the supercomplex. The largest clade-specific subunit, ApiCox13, contains a CDGSH-type iron–zinc finger domain on the matrix side, with some residues contributing to cardiolipin binding (Extended Data Fig. 4). As this protein fold is known to have evolved varying metal binding capacities (Zn^2+^ or Fe_2_S_2_)^18^, we performed sequence alignments and structure prediction of ApiCox13 homologs. This analysis indicated that Fe_2_S_2_ sites are well conserved in Apicomplexa, while homologs from marine species in related phyla instead contain tetrahedrally coordinated zinc (Extended Data Fig. 4). These findings are in line with the observed essentiality of Fe_2_S_2_ binding capability for CIV integrity and parasite survival in *T.* *gondii*^19^.

**Extended Data Fig. 5:**
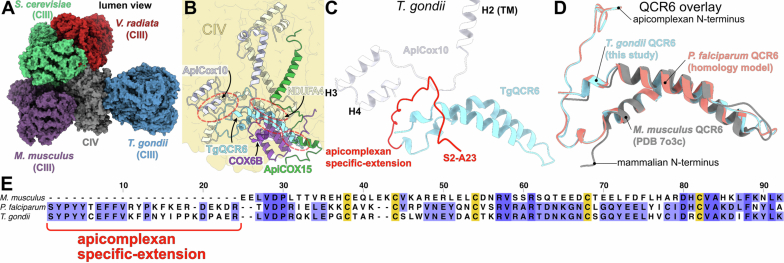
Diversity of mitochondrial respiratory chain supercomplexes architecture in different organisms and the apicomplexan-conserved subunits and extensions that establish the divergent III_2_-IV interface in *T. gondii*. **(a)** Overlay of supercomplex structures aligned on CIV highlights different architectures (luminal view), as revealed by differing positions of CIII from mammals (*M. musculus*, PDB 7o37 mature supercomplex), yeast (*S. cerevisiae*, PDB 6giq) and plant (*V. radiata*, 7jrp). The mammalian and *T. gondii* CIII homologs bind to opposite sides of CIV. **(b)** TgQCR6 (hinge protein) interaction with four contact sites on CIV highlighted. **(c)** *T. gondii* III_2_-IV interface (this study) of TgQCR6 with ApiCox10 involving contacts via an apicomplexan-conserved N-terminal extension. **(d-e)** Structural overlay (**D**) and structure-based multiple sequence alignment (**E**) of the murine, *P. falciparum* and *T. gondii* QCR6 homologs showing a conserved hairpin structure. The N-terminal QCR6 extension that interacts with ApiCox10 is also found in *P. falciparum*. The mammalian N-terminus is cleaved as part of a mitochondrial targeting sequence.

The architecture of the *T.* *gondii* supercomplex is unique among all previously described respiratory supercomplexes and this is mediated by a series of clade-specific subunits and extensions. When compared with the mature mammalian supercomplex III_2_–IV, the *T.* *gondii* CIV is found in a similar position relative to CIII, but rotated by ~180° (Extended Data Fig. 5a), therefore interacting with a different set of subunits. This leads to a unique relative positioning of *T.* *gondii* complexes III and IV, compared with mammalian, yeast and plant supercomplexes^20–22^ (Fig. 2f and Extended Data Fig. 5a), indicating that non-opisthokont lineages may represent most of respiratory supercomplex diversity. Whereas the mammalian assembly factor SCAF1 ties CIV to CIII in proximity to QCR6, TgQCR6 is facing toward NDUFA4 and the clade-conserved subunits, ApiCox10 and 15, located on the opposite side of CIV, thus generating a unique interface and III_2_–IV supercomplex architecture (Fig. 2g and Extended Data Fig. 5b). Furthermore, owing to the kinked architecture of the *T.* *gondii* supercomplex, CIV is associated to CIII only via the conserved TgQCR6, forming lumenal interactions with the four CIV subunits COX6B, ApiCox10, NDUFA4 and ApiCox15 (spheres in Fig. 2h). The interaction with ApiCox10 is mediated via an apicomplexan-conserved N-terminal extension (residues 2–23) of TgQCR6, which forms hydrophobic interactions with a helix hairpin (H3–H4) of ApiCox10 (Extended Data Fig. 5c). ApiCox15 contains a single TM-helix and a 38-residue lumenal region that contacts the TgQCR6 helix hairpin. Finally, unlike its mammalian homolog, the apicomplexan NDUFA4 C-terminus contains a structured horizontal helix that interacts with the TgQCR6 hairpin (Fig. 2g,h,i) on the lumenal side. The clade-specific interacting subunits and phylum-specific protein extensions are conserved in the malaria-causing *Plasmodium falciparum* (Extended Data Fig. 5d,e) and other apicomplexans, indicating that the newly described III_2_–IV interface and unusual supercomplex architecture are probably conserved in apicomplexan parasites.

**Fig. 3:**
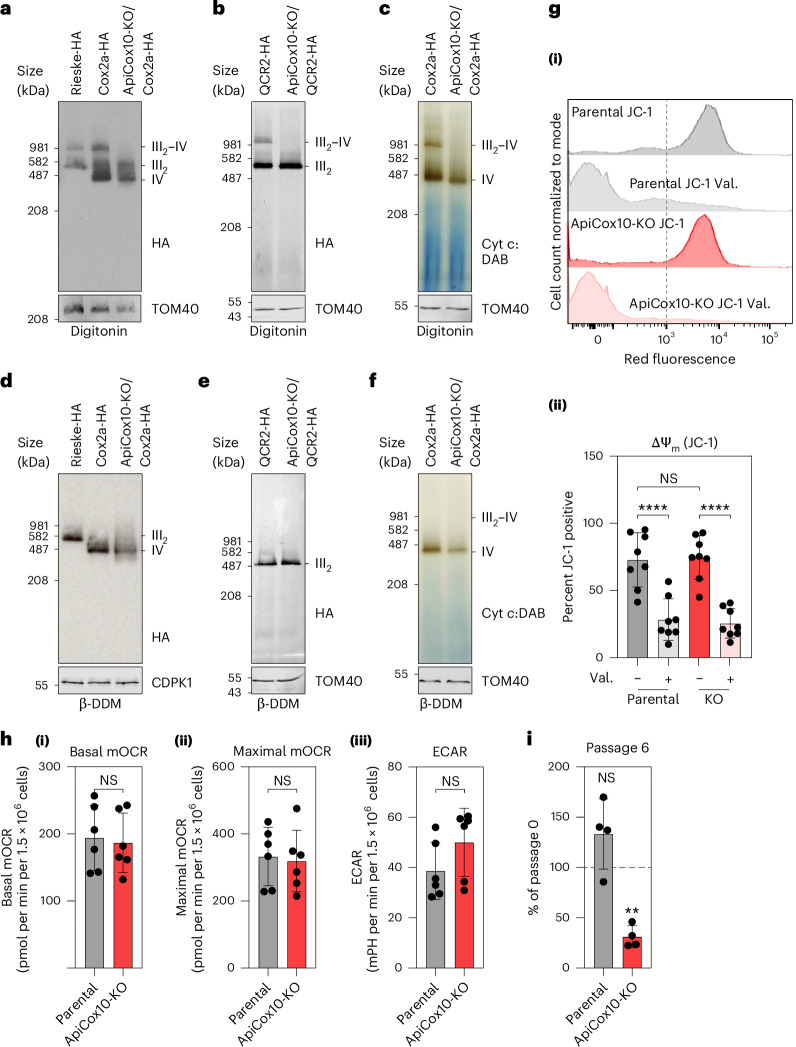
Supercomplex assembly is critical for *T. gondii* fitness. **a**, Native PAGE analysis of Rieske-HA (CIII_2_ tagged line), Cox2a-HA (CIV tagged line) and ApiCox10-KO in the Cox2a-HA background (ApiCox10-KO/Cox2a-HA). Total lysates were treated with digitonin and separated by BN–PAGE, followed by immunoblot analysis with anti-HA antibodies, as well as anti-TOM40 as a loading control. Positions of complexes are indicated. **b**, Native PAGE analysis of QCR2-HA (CIII_2_ tagged line) and ApiCox10-KO in the QCR2-HA background (ApiCox10-KO/QCR2-HA) treated with digitonin, as in **a**. Samples were also separated by SDS–PAGE and immunoblot analysis with anti-TOM40 antibodies performed as a loading control. **c**, Native PAGE analysis of Cox2a-HA ApiCox10-KO/Cox2a-HA treated with digitonin, followed by cytochrome *c* DAB staining to visualize CIV activity. **d**–**f**, Native PAGE analysis of the lines in **a** (**d**), **b** (**e**) and **c** (**f**) extracted using β-DDM. **g**, Measurement of mitochondrial membrane potential using JC-1 dye via flow cytometry analysis. (i): *T. gondii* stained with the dye JC-1 indicated that both lines possess a mitochondrial membrane potential that is sensitive to the ionophore valinomycin. The population to the right of the dotted gray line is JC-1 positive. (ii): quantification of mitochondrial membrane potential by population that is positive for JC-1 staining. Graphs show mean ± s.d., from eight independent experiments. One-way ANOVA followed by Tukey’s multiple pairwise comparisons was performed, and *P* values from relevant pairs are displayed. ****P < 0.0001. **h**, Extracellular flux analysis of (i) basal mitochondrial OCR, (ii) maximal mitochondrial OCR and (iii) ECAR of parental and ApiCox10-KO parasites. Graphs show mean ± s.d. from six independent experiments. *P* value was determined from a two-tailed unpaired Student’s *t*-test. **i**, A mixed culture growth competition assay of ApiCox10-KO or parental mNEON fluorescent parasites with tdTomato parasites. Relative abundance (compared with passage 0) of ApiCox10-KO or parental parasites after six passages. Points are mean of four independent experiments, error bars are s.d. *P* value was determined using a two-tailed one-sample *t*-test comparing values to passage 0, ** *P* = 0.0011. NS, not significant. Source data.

**Extended Data Fig. 6:**
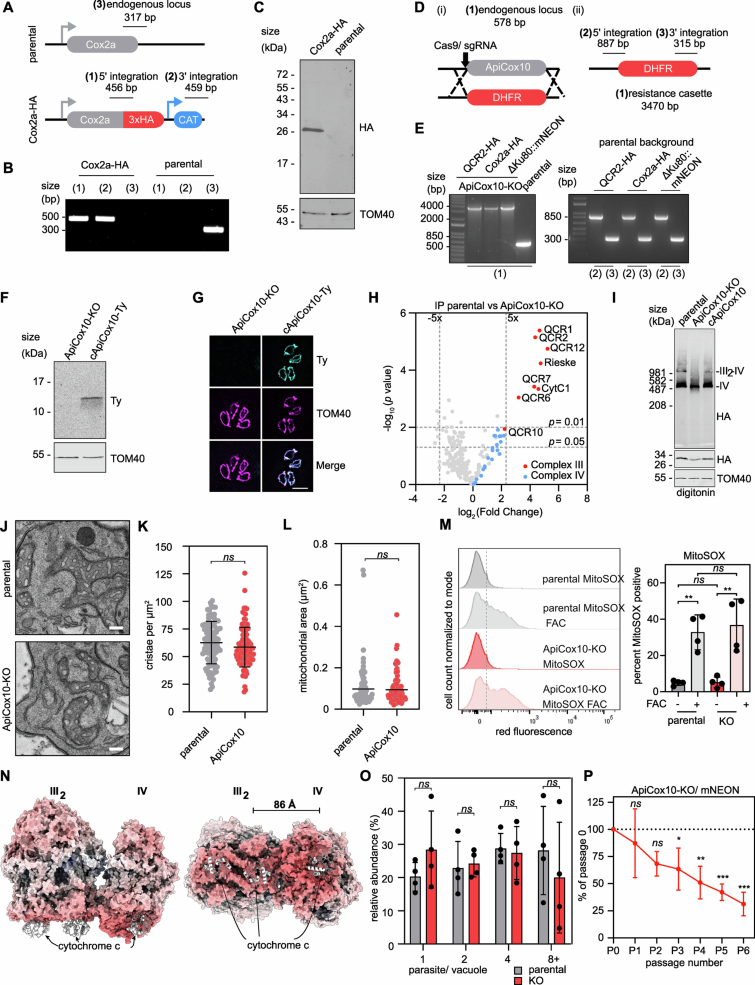
Generation and characterization of ApiCox10-KO line. **(a)** Schematic of the strategy used to C-terminally HA-epitope tag the Cox2a protein. The expected size of integration PCRs are shown. **(b)** PCR to test integration of HA-epitope tag and CAT selection cassette into the endogenous locus, as outlined in *(A)*. **(c)** Immunoblot analysis of whole cell lysate extracted from Cox2a-HA and parental parasites. Samples were separated by SDS-PAGE, blotted, and detected using anti-HA and anti-TOM40 as a loading control. **(d)** Schematic of the strategy used to replace the coding sequence of ApiCox10 with a DHFR selection cassette. The expected size of integration PCRs are shown. **(e)** PCR to test integration of the DHFR selection cassette into the endogenous locus, in Cox2a-HA, QCR2-HA and mNEON::Δ*ku80* parental background, as outlined in (D). **(f)** Immunoblot analysis of ApiCox10-KO and complementation line (cApiCox10-Ty) parasites, detected using anti-Ty and anti-TOM40. **(g)** Immunofluorescence assay of ApiCox10-KO and cApiCox10-Ty parasites, labeled with anti-Ty and anti-TOM40. Scale bar is 5 μM. **(h)** Volcano plot of proteomic data from Cox2a-HA vs ApiCox10/Cox2a-HA immunoprecipitations, showing the -log10 *P* values and the log2 fold changes of proteins detected by mass spectrometry in 3 or more replicates, from 4 independent experiments. *P* values were calculated using a two-tailed t-test. Horizontal dotted gray line denotes *P*= 0.05 and *P* = 0.01. Vertical dotted line denotes 5x enrichment. Complex III subunits are labeled in red and complex IV subunits are labeled in the blue. **(i)** Native-PAGE and immunoblot analysis of Cox2a-HA, ApiCox10-KO/ Cox2a-HA and the complemented cApiCox10-Ty lines. Total lysate was treated with digitonin and separated by BN-PAGE, followed by immunoblot analysis. Positions of complexes are indicated. **(j)** Transmission electron microscopy (TEM) of parasites to visualize mitochondrial cristae. Scale bar is 200 nm. **(k,l)** Quantification of number of cristae cross sections per mitochondrial surface area (K) and mitochondrial area (L) in parental and ApiCox10-KO parasites from TEM. Data points from 100 mitochondrial profiles, with mean shown ( ± s.d. in K). *P* value from a two-tailed unpaired t*-*test. **(m)** MitoSOX labeling to detect mitochondrial ROS at steady state or upon treatment with ferric ammonium chloride (FAC) in ApiCox10 knockout and parental parasites in mNEON background via flow cytometry analysis. Left: red fluorescence of *T. gondii*, in the absence or presence of ferric ammonium chloride (FAC), stained with MitoSOX. Population to the right of the dotted gray line are MitoSOX positive. Right: Quantification of population that is positive for MitoSOX signal. Graphs show mean ± s.d., from 4 independent experiments One-way ANOVA followed by Turkey’s multiple pairwise comparisons was performed, and *P* value from relevant pairs displayed. ns, no significant difference; ** *P* < 0.01 (parental vs parental + FAC *P* = 0.0037; KO vs KO + FAC *P* = 0.0015). (**n**) Surface electrostatics of the III2-IV (calculated with the Adaptive Poisson-Boltzmann Solver, APBS4) reveals a negative lumenal patch. Cytochrome-c binding sites were inferred from overlays of PDBs 5iy5 and 3cx5. **(o)** Quantification of number of parasites per vacuole for parental and ApiCox10-KO parasites. Error bars are mean ±s.d. from 4 independent experiments, for which over 250 vacuoles were counted for each replicate. The *P* value determined by multiple two-tailed t-tests with a Holm-Sidak correction applied. **(p)** Mixed culture growth competition assay of ApiCox10-KO or parental mNEON fluorescent parasites with tdTomato parasites. Relative abundance (compared to passage 0) of ApiCox10-KO parasites across 6 passages. Points are mean ± s.d., from 4 independent experiments, *P* value determined from a one-way ANOVA, corrected for multiple comparisons (Dunnett) (ns, no significant difference; P3 *P* = 0.0242; P4 *P* = 0.0022; P5 *P* = 0.0004; P6 *P* < 0.0001). Source data

Given the observed interactions of the clade-specific ApiCox10 and ApiCox15 with TgQCR6 at the supercomplex interface, we hypothesized that deletion of either of those proteins would result in supercomplex disassembly, allowing us to address the role of supercomplex formation. We attempted to generate ApiCox10 and ApiCox15 individual knockouts (KOs) using a clustered regularly interspaced short palindromic repeats (CRISPR)–Cas9 system. Five independent transfections and a screen of 50 clones failed to isolate an ApiCox15-KO mutant suggesting that this gene is refractory for full deletion. On the other hand, we were able to generate an ApiCox10-KO in two separate background lines, one where the CIV subunit Cox2a is endogenously tagged with a C-terminal hemagglutinin (HA) tag, and another where the CIII subunit QCR2 is endogenously HA tagged^8^. Additionally, we generated a complemented line where a Ty-tagged ApiCox10 is reintroduced (Extended Data Fig. 6a–g). Native gel electrophoresis analysis demonstrated the loss of III_2_–IV supercomplexes in both ApiCox10-KO lines (Fig. 3a–c), and this was further confirmed via proteomics analysis of Cox2a-HA immunoprecipitation (Extended Data Fig. 6h and Supplementary Data 1). Importantly, the individual CIV and CIII_2_ remain intact (Fig. 3d–f) and the Ty-tagged ApiCox10 complementation restored the supercomplex (Extended Data Fig. 6i), confirming the unique interface architecture of the apicomplexan III_2_–IV supercomplex and its dependence on a clade-specific subunit for its assembly.

**Extended Data Fig. 7:**
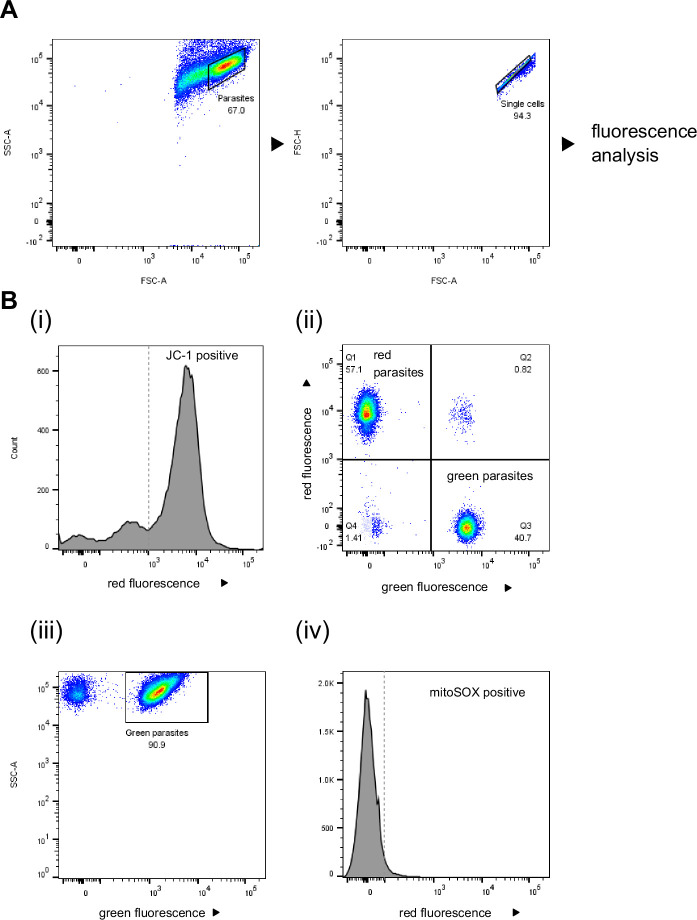
Gating strategy for flow cytometry. **(a)** Example gating strategy for to isolate parasites for flow cytometry analysis for Fig. 3g,i and Extended Data Fig. 6M,P. Gates were drawn as indicated manually using FlowJo software, using forward and side scatter plots (SSC-A vs FSC-A) to gate on the parasite population and separate it from host cell debris. Single cells were then selected (FSC-A vs FSC-H). Parasites were then analyzed for fluorescence. **(b)** Flow cytometry fluorescence analysis (i) analysis of red fluorescence for JC-1 assay to determine mitochondrial membrane potential in Fig. 3g (left) (ii) analysis of green and red fluorescence for JC-1 assay to determine mitochondrial membrane potential in Fig. 3g (right). Comparison of parasites showing red and green or green only fluorescence was made, with positions of quadrants determined by unstained and valinomycin controls. (iii) analysis of green and red fluorescent parasites for growth competition assay in Fig. 3i and Extended Data Fig. 6P. Green and red parasite quadrants determined by analyzing mNEON::*Δku80* and tdTomato::*Δku80* parental parasites separately (iv) analysis of red fluorescence for MitoSOX assay to determine mitochondrial ROS generation in Extended Data Fig. 6M.

The supercomplex structure raises the question of whether the observed kinked architecture contributes to the induction of membrane curvature or whether it has evolved to allow accommodation in the unique bulbous mitochondrial cristae found in apicomplexans. Analysis of mitochondria in electron microscopy thin sections of the ApiCox10-KO line pointed to unchanged cristae morphology and density, in support of the latter scenario (Extended Data Fig. 6j–l). Likewise, ApiCox10-KO had no impact on the mitochondrial redox state (Extended Data Fig. 6m). Furthermore, examination of the mitochondrial membrane potential and oxygen consumption rate (OCR) in extracellular ApiCox10-KO, as proxy measurements for respiratory activity in tachyzoites, revealed no alteration compared to the parental line (Fig. 3g,h and Extended Data Fig. 7). This finding is in stark contrast to *Saccharomyces cerevisiae*, where supercomplex disruption impairs electron transfer efficiency in a cytochrome *c* level-dependent manner^4^. Interestingly, analysis of the surface charges on the *T.* *gondii* supercomplex shows a continuous negatively charged patch on the lumen region (Extended Data Fig. 6n), which is conserved in the yeast supercomplex, where it has been suggested to facilitate intracomplex cytochrome *c* transfer by two-dimensional (2D) diffusion^23^. Together, our findings suggest that cytochrome *c* diffusion does not limit respiration in *T.* *gondii* tachyzoites.

Finally, we tested whether the inability to form respiratory supercomplexes inflicts a fitness cost on the parasite. Analysis of parasite replication revealed a potential delay in reaching eight parasites per vacuole (Extended Data Fig. 6o). We thus created an ApiCox10-KO in a fluorescent background line and performed a growth competition assay. ApiCox10-KO parasites grown in the same culture as wild-type parasites were consistently outcompeted, indicating decreased fitness (Fig. 3i and Extended Data Figs. 6p and 7). Overall, parasites that are unable to form supercomplexes have no observable defects in respiratory activity, but display a fitness penalty, indicating that supercomplexes play an important fitness-conferring role in mitochondrial function that is not directly related to primary catalytic function. The finding that clade-specific subunits, which mediate supercomplex formation are important for competitive fitness may indicate that they convey increased stability or aid localization of the kinked supercomplexes to the uniquely shaped cristae (as opposed to the inner boundary membrane) to enable efficient formation of a membrane potential. The fitness defects associated with loss of supercomplexes in a tachyzoite competition assay suggest that supercomplex functions may become more relevant and observable in vivo or in different stages of the *T.* *gondii* life cycle.

## The structural basis for parasite-specific atovaquone binding

CIII (cytochrome *bc*_1_ complex) plays a crucial role in electron transfer and proton pumping. The pumping of protons into the cristae lumen occurs via the Q-cycle, which involves sequential ubiquinol oxidation and ubiquinone reduction. Both reactions occur in specific quinone binding sites within the cytochrome *b* (Cyt-b) subunit, called Q_o_ (oxidation) and Q_i_ (reduction), which are essential for catalytic activity. Atovaquone is a Food and Drug Administration-approved antimalarial drug that competitively inhibits the Cyt-b Q_o_ site and is also effective against toxoplasmosis^24^ (EC_50_ of 138 nM (ref. ^25^)). However, resistance to atovaquone has led to the development of novel inhibitors, such as ELQs, which target the Q_i_ site^25^. The basis of selectivity of inhibition at either site is not fully understood.

The current understanding of species-specific Q_o_ site inhibition by atovaquone is deduced from homology models of CIII structures of other species^26^, but experimental structures from apicomplexans have not been determined. Moreover, ELQ-300 is a Q_i_ site inhibitor undergoing late-stage preclinical testing as a new antimalarial drug in the Medicines for Malaria portfolio, which is effective against blood, liver and mosquito-stage malaria, and also displays efficacy against *T.* *gondii* and other apicomplexan pathogens^27,28^. When used in combination therapy with atovaquone, ELQ-300 is highly synergistic in a murine malaria model^29^. However, the atovaquone/ELQ-300-bound structure of CIII has not been reported, and the structural basis for parasite-specific Cyt-b inhibition is not known.

**Fig. 4:**
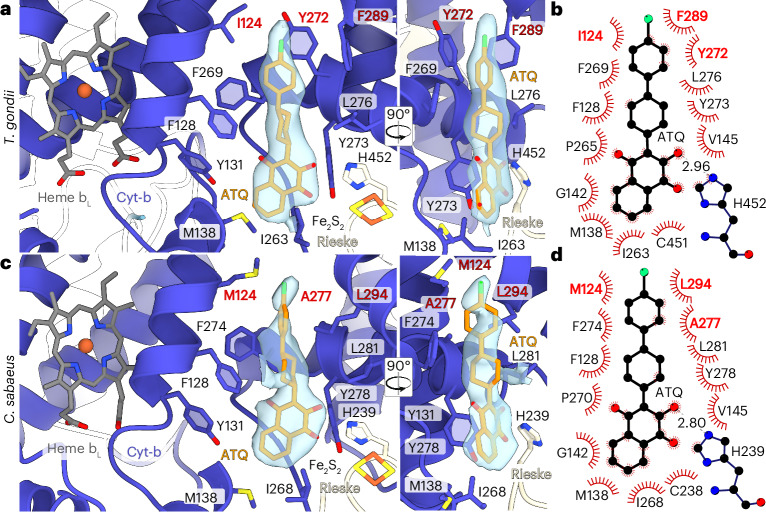
Atovaquone-bound structures of the *T.* *gondii* and mammalian CIII reveals the structural basis for species-specific Q_o_ site binding. **a**, View of the Q_o_ site of *T.* *gondii* occupied by atovaquone, which prevents electron transfer to heme b_L_ and Fe_2_S_2_ in the lumenal domain of the Rieske subunit, which occupies the b-state. Residues responsible for apicomplexan-specific atovaquone sensitivity are shown in red. **b**, A ligand diagram of atovaquone interactions in the *T.* *gondii* Q_o_ site. **c**, View of the Q_o_ site of *C.* *sabaeus* occupied with atovaquone. **d**, A schematic of atovaquone interactions in the *C.* *sabaeus* Q_o_ site.

**Extended Data Fig. 8:**
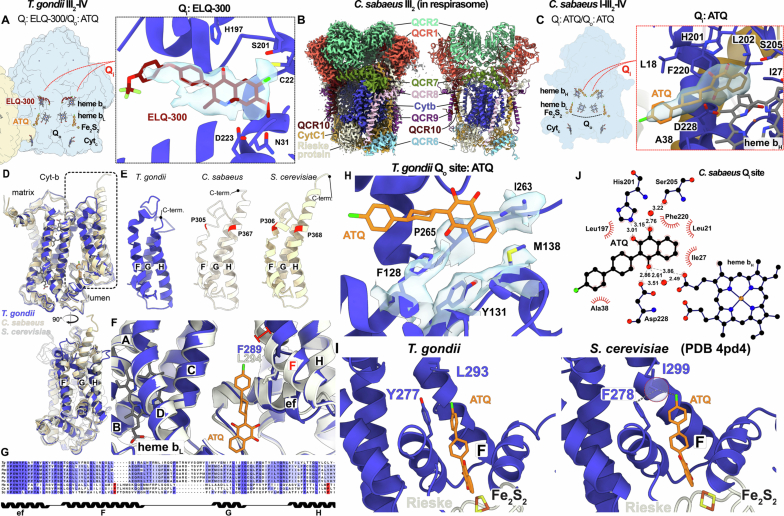
Structural divergence of the apicomplexan cytochrome-b and the different mode of inhibition of the parasite and host by atovaquone. **(a)** *T. gondii* supercomplex structure with heme molecules and inhibitors (ELQ-300 and atovaquone (ATQ)) **(b)** Cryo-EM map (left) and model (right) of the *C. sabaeus* CIII. **(c)** Schematic of the *C. sabaeus* CIII with heme groups and two ATQ copies (orange) per monomer shown. Inset depicts close-up view of the Q_i_ site occupied with atovaquone. **(d)** Overlay of Cyt-b structures from *T. gondii*, *C. sabaeus* (both this study) and *S. cerevisiae* with the module of helices F-H highlighted. **(e)** Comparison of helices **F**,**G**,**H**. The *T. gondii* helices F and H are straight and lack the conserved proline residues (red) found in the opisthokont homologs. **(f)** Overlay of the atovaquone-inhibited Cyt-b structures from *T. gondii* and *C. sabaeus* showing that the kink in the mammalian helix F causes it to extend further into the Q_o_ site. Two affected residues interacting with ATQ are shown. **(g)** Multiple sequence alignment showing the absence of conserved proline residues (red) in helices F and H. *Tg*, *Toxoplasma gondii*; *Pf*, *Plasmodium falciparum*; *Pm*, *Plasmodium malariae*, *Pk*, *Plasmodium knowlesi*; *Pv*, *Plasmodium vivax*; *Po*, *Plasmodium ovale*; *Hs*, *Homo sapiens*; *Cs*, *Chlorocebus sabaeus*; *Sc*, *Saccharomyces cerevisiae*. **(h)** *T. gondii* Q_o_ site with ATQ and surrounding conserved residues (map in blue). **(i)** Comparison of atovaquone bound Q_o_ sites in *T. gondii* (left) and *S. cerevisiae* (right; PDB 4pd4). **(j)** Ligand diagram of atovaquone in the *C. sabaeus* Q_i_ site.

We determined the structure of the *T.* *gondii* III_2_–IV supercomplex in the presence of atovaquone and ELQ-300 (10 µM each) at 2.8 Å resolution. The cryo-EM map shows both ELQ-300 (Q_i_) and atovaquone (Q_o_) bound, allowing insight into the binding mode of the two inhibitors (Fig. 4a,b and Extended Data Fig. 8). As our mitochondria preparations of the obligate intracellular parasite *T.* *gondii* also contained substantial amounts of mitochondria from the host cells (‘Vero’ cells from the African green monkey, *Cholorocebus sabaeus*), the final cryo-EM sample contained a mixture of mitochondrial complexes from both species, which we classified computationally (Extended Data Fig. 1). From the same cryo-EM dataset, we thus also determined the structure of CIII within the co-purified *Cholorocebus sabaeus* I–III_2_–IV respirasome at 2.8 Å resolution (with atovaquone bound in Q_i_ and Q_o_ sites, see below), allowing direct comparison of architecture and inhibitor binding between parasite and host (Extended Data Table 1 and Extended Data Fig. 1). In our structure, *Tg*Cyt-b displays the conserved fold including eight transmembrane helices (A–H) and conserved redox centers (Extended Data Fig. 8). Notably, helices F, G and H are substantially remodeled. In opisthokonts (which includes yeast and mammals), helix F is curved and contains a 3_10_ helix that introduces a bend in the transmembrane segment and is followed by a conserved proline residue (P305 in humans and *C.* *sabaeus*) that acts as a helix breaker. This conserved proline residue is missing in apicomplexan Cyt-b homologs, resulting in a straight α-helical helix F. Likewise, the *T.* *gondii* FG-loop (N306–W324, human 309–315) is remodeled, followed by a shortened hairpin of helices G and H. These features affect the positioning of residues that interact with the chlorophenyl moiety of atovaquone.

The binding pocket for the hydroxynaphthoquinone group of the inhibitor is conserved between *C.* *sabaeus* and *T.* *gondii*, with our structure revealing interactions with residues F128, Y131, P265, M138 and I263 (Fig. 4), of which the latter two were previously found mutated in atovaquone-resistant *T.* *gondii* lines^30^. The difference in Q_o_ site affinity arises from a group of parasite-specific residues (I124, Y272 and F289) that interact with the chlorophenyl moiety of atovaquone. Among these, Y272 (*T.* *gondii* numbering) on the EF-helix is occupied by an alanine in higher primates (A277 in humans and *C.* *sabaeus*). In the inhibited Cyt-b structure from *S.* *cerevisiae*, the chlorophenyl and cyclohexane rings of atovaquone are modeled in a near parallel configuration^26^. Our structure reveals that in *T.* *gondii*, the two moieties are instead oriented orthogonally, leading to a pi-stacking interaction of the chlorophenyl group with Y272 (Fig. 4). The aromatic character of this residue is also conserved in *Plasmodium* species causing malaria in humans (F267). Indeed, the single mutations F267V and F267I convey atovaquone resistance in *P.* *falciparum* and *P.* *yoelii*, respectively^31,32^, indicating that aromatic interactions, rather than unspecific hydrophobic contacts, are required for atovaquone binding. Our structure reveals that Y272 acts to position the chlorophenyl group of atovaquone to convey parasite-specific ligand interactions.

The remodeled *Tg*Cyt-b structure affects the binding of atovaquone in the Q_o_ site. Owing to the curved helix F structure, in the *S.* *cerevisiae* Cyt-b, the I299 sidechain is partially inserted between the respective aromatic residue (F278) and the chlorophenyl group, preventing a stacking interaction as observed in our *T.* *gondii* structure (Extended Data Fig. 8e). This may explain the potential difference in sensitivity between yeast^26,33^ and *P.* *falciparum*^27,34^.

Notably, the binding of atovaquone to the Q_o_ site generates an induced fit. Comparison to our *T.* *gondii* structure with unoccupied Q_o_ site (see below), revealed that the EF loop and cd1 helix undergo movements to widen the pocket. In this process, I263 on the EF loop, which would result in a clash with atovaquone in the unoccupied state, moves by 2 Å to accommodate the ligand (Supplementary Movie 1).

While the atovaquone EC_50_ value is in the low nanomolar range for both *P.* *falciparum* and *T.* *gondii* cytochrome *bc*_1_^35,36^, the EC_50_ for the mammalian homolog is more than 100 times higher^27^ (EC_50_ *T.* *gondii* of 138 nM, 38 µM human foreskin fibroblasts (HFF)^25^). Remarkably, we also found atovaquone bound not only in the Q_o_ site, but also the Q_i_ site of the *C.* *sabaeus* Cyt-b, which is part of the I–III_2_–IV supercomplex. Our structure indicates that in the dual-site inhibited mammalian Cyt-b, the atovaquone molecules of the Q_i_ and Q_o_ sites probably adopt two different tautomers to enable the formation of hydrogen bond networks in the two quinone binding sites (Extended Data Fig. 8 and Supplementary Discussion). This observed dual atovaquone inhibition of the mammalian complex (Q_i_ + Q_o_) indicates that ELQ-300 binds the mammalian Q_i_ site very weakly or not at all. Thus, by co-determining the structures of both the mammalian and *T.* *gondii* CIII in the presence of equimolar atovaquone/ELQ-300 concentrations (10 µM) from the same heterogeneous sample and revealing their different drug binding, we provide structural evidence for the high parasite selectivity of ELQ-300 as a Q_i_-site inhibitor^27^.

## The Q_i_ inhibitor ELQ-300 adopts an unexpected binding pose

**Fig. 5:**
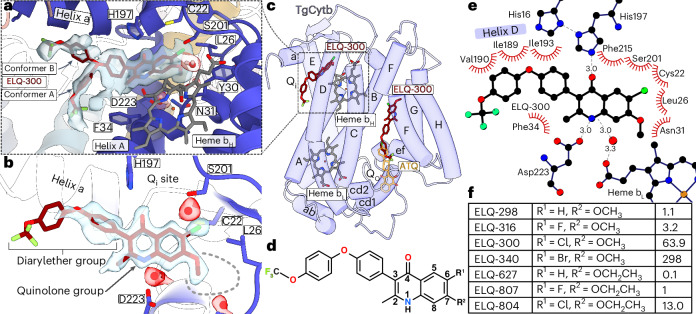
An unexpected Q_i_ binding pose of ELQ-300 allows structure-guided design of inhibitors with increased potency. **a**, View of ELQ-300 bound in the Q_i_ site of *T.* *gondii*, showing both conformer A and B. **b**, A close-up view of conformer B of ELQ-300 bound in the Q_i_ site. Parasite-specific aqueous pocket denoted by gray-dashed line. **c**, *Tg*Cyt-b with heme and ELQ-300 binding sites. Helices A–H are connected by loops including AB, CD and EF, with interspersed horizontal helices a, ab, cd1, cd2 and ef. The overlapping atovaquone site (Q_o_) is shown in transparent orange. The dashed line shows the close-up region depicted in **a**. **d**, The structure formula of ELQ-300. **e**, Ligand diagram of ELQ-300 in the Q_i_ site highlighting hydrogen bonds and hydrophobic interactions. **f**, Name, formula and EC_50_ values (nM) of the ELQs that were tested.

**Extended Data Fig. 9:**
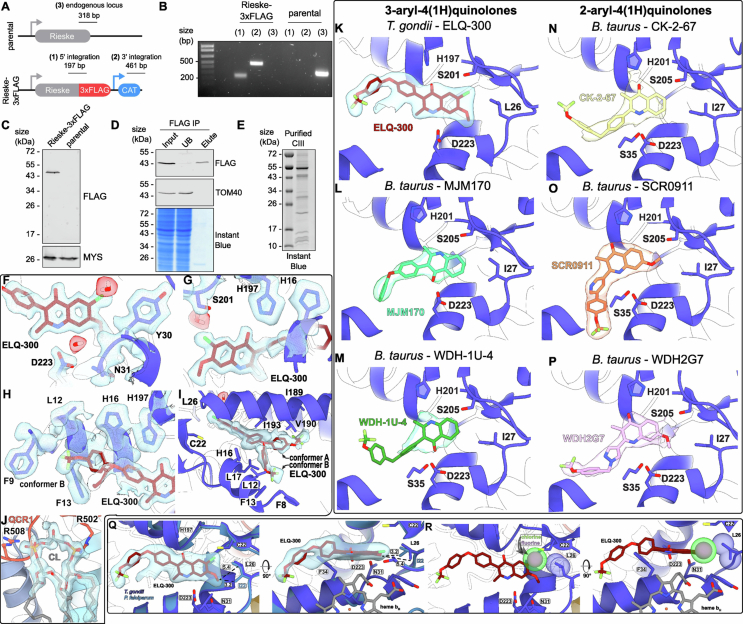
Cryo-EM structure of the affinity-purified *T. gondii* complex III, obtained to elicit the binding mode of ELQ-300 at the Q_i_ site reveals critical differences to quinolone-bound bovine structures. **(a)** Schematic of the strategy used to C-terminally triple FLAG-epitope tag the Rieske protein. The expected size of integration PCRs are shown. **(b)** PCR to test integration of FLAG-epitope tag and CAT selection cassette into the endogenous locus, as outlined in *(A)*. **(c)** Immunoblot analysis of whole cell lysate extracted from Rieske-3xFLAG and parental parasites. Samples were separated by SDS-PAGE, blotted, and detected using anti-FLAG and anti-MYS as a loading control. **(d)** Immunoblot analysis of fractions from FLAG immunoprecipitation. Equal cell equivalents of input, unbound (UB) and elute fractions were separated by SDS-PAGE and either stained with instant blue or blotted, and detected using anti-FLAG and anti-TOM40. **(e)** Purified complex III after one round of gel filtration: 5 µg of protein separated by SDS-PAGE and stained with instant blue. **(f-j)** Cryo-EM densities (transparent blue) for residues surrounding the Q_i_ site showing the ELQ front view (**F**), back view (**G**), closeup on the cytochrome-b N-terminus (**H**) and conformers A and B of ELQ (**I**), closeup of a cardiolipin molecule (**J**). **(k)** In *T. gondii* (this study) the carbonyl and amino groups interact with H197 and D223, respectively. **(l-m)** In X-ray structures of the *B. taurus* homolog, the orientation of the 3-aryl quinolones is rotated by 180 degrees, leading to a different position of the diaryl-rest when compared to the parasite structure (L: PDB ID 5NMI, ref. ^68^; M: 6ZFS). Shown are inhibitors MJM170 (L) and WDH-1U-4 (M). **(n-p)** By comparison, owing to a flipped quinolone orientation 2-aryl-quinolones bind with the diaryl-like group facing in the same direction as in other structures of the bovine enzyme (N: PDB ID 7R3V; O: 5OKD; P: 6HAW), shown are inhibitors CK-2-67 (**N**), SCR0911 (**O**) and WDH2G7 (**P**). **(q-r)** Species-specific mutations in the apicomplexan Q_i_ site explain susceptibility to ELQ-300. **(q)** ELQ-300 bound in the *T. gondii* cytochrome-b (this study, cryo-EM density in transparent blue) with overlaid a predicted model of the *P. falciparum* homolog (AlphaFold2). The apparent favorable interaction of the *P. falciparum* I22 with the methoxy group is indicated. (**r)** Van der Waals atomic radii of the 3-position chlorine and L26 shown in comparison with the smaller fluorine atom found in ELQ-316. Source data

To reveal its binding mechanism at high resolution, we affinity-purified the *T.* *gondii* CIII embedded in amphipols via the 3xFLAG-tagged Rieske subunit (Extended Data Fig. 9a–e), inhibited by ELQ-300 only, and determined its cryo-EM structure to 1.8 Å resolution (Extended Data Fig. 1 and Extended Data Table 1). Surprisingly, the C_2_-symmetric CIII structure contains two bound ELQ-300 molecules per Cyt-b monomer, one at the Q_i_ site and, unexpectedly, another in the central cavity of the Q_o_ channel (Fig. 5a–d). In the Q_i_ pocket, ELQ-300 displays an unexpected binding mode, which is well supported by our cryo-EM density (Fig. 5b and Extended Data Fig. 9f–h). The 4-(1H)-quinolone group is pinned by hydrogen bonds with H197 and D223, interacting with ketone and amino groups of the ligand at N–O distances of 3.0 Å, respectively (Fig. 5a and Extended Data Fig. 9f–g). The ketone group of ELQ-300 also interacts with the heme *b*_H_ propionate group via an ordered water (Fig. 5a,e). This binding mode differs markedly from the orientation of other 3-diaryl 4-(1H)-quinolones in the Q_i_ site of the bovine enzyme, which was found to be rotated 180° (ref. ^37^) (Extended Data Fig. 9l–m). Owing to the fixed orientation of the quinolone group in our *T.* *gondii* structure, the diarylether group in position-2 extends toward helix D (on the matrix side), which is markedly different from the binding pose observed in the bovine homolog, in which quinolones with both 2- or 3-diaryl-groups extend in the opposite direction, toward helix A (in the lumen side; Fig. 5c,e and Extended Data Fig. 9k–p). This finding is similar to the binding mode of atovaquone in the Q_i_ site observed in our *C.* *sabaeus* structure, in which the chlorophenyl-cyclohexyl group also points toward helix A, indicating a similar binding mode to that seen for other inhibitors of the mammalian Q_i_ site^26,29,30^. In our *T.* *gondii* structure, the orientation of the first aryl group is determined by the adjacent I193 on helix D, leading to a 63° angle between the aryl and quinolone planes. The peripheral trifluoro-methoxy-phenoxy group displays flexibility and adopts two distinct conformations (Fig. 5a). Conformer A makes contacts along helix D with I189, V190 and I193, whereas through a rotation around the ether bond, conformer B extends toward helix a to interact with a set of hydrophobic residues (Extended Data Fig. 9i). The hydrophobic character of the Q_i_ site entrance is probably enhanced by the specific recruitment of an adjacent cardiolipin molecule (Extended Data Fig. 9j).

One conspicuous residue that may contribute to determining the binding mode of ELQ-300 is F34 from helix A, which interacts with the methyl group in the 2-position (Fig. 5a,e). This residue is conserved in *P.* *falciparum*, but not in human (S35) and it is thus possible that its bulky character contributes to dictating the orientation of the 3-aryl quinolones in apicomplexan parasites by restricting the entrance of the Q_i_ site. Thus, our structure reveals an unexpected binding mode of the ELQ-300 in the apicomplexan Q_i_ site, which differs markedly from previously determined structures of the inhibited mammalian CIII and provides an accurate molecular model for developing antimalarial inhibitors.

Our data also reveal the structural basis for the observed parasite selectivity of ELQ-300 for the Q_i_ site^35^^,^^38^^,^^39^. In the deepest part of the *T.* *gondii* Q_i_ pocket, L26 interacts with the chloro and methoxy groups of the quinolone (Extended Data Fig. 9q). This finding corroborates the observation that an I22L mutation of the respective residue in *P.* *falciparum* (clone D1) causes a 24-fold reduction in sensitivity to ELQ-300 (ref. ^40^). L26 further contacts the chlorine substituent at the van der Waals distance (Extended Data Fig. 9r), suggesting it is important for ligand positioning within the Q_i_ site. This finding agrees with previous evidence showing that the size of the atomic radius of the 6-position substituent, fluorine (ELQ-316) or hydrogen (ELQ-298), is inversely correlated to potency against *T.* *gondii*^25,28^. Furthermore, our structure indicates that species-specific affinity may not be solely owing to steric hindrance introduced by L26, but due to additional contacts of the 3′-methyl group of the *P.* *falciparum* I22 sidechain with the 7-methoxy group of ELQ-300 (Extended Data Fig. 9q). The methoxy-substituent at the 7-position is known to limit Q_i_ site affinity in the mammalian host^25,41^.

## Structure-based design of 7-ethoxy ELQs with increased potency

On the basis of the above observation, the EC_50_ of various ELQs decreases together with the atomic radius in position 6 (Br > Cl > F > H). We concluded that this may be owing to a deeper penetration of the inhibitor into the Q_i_ site, allowing a stronger interaction with the substituent at the position 7 (Extended Data Fig. 9r). The EC_50_ of ELQ-340, ELQ-300, ELQ-316 and ELQ-298 were 238 nM (95% confidence interval (CI) 215, 261), 63.9 nM (95% CI 53–74.9), 3.2 nM (95% CI 2.7–3.6) and 1.1 nM (95% CI 0.5–1.7), respectively (Extended Data Table 1). Indeed, an analysis of the apicomplexan Q_i_ site revealed that a parasite-specific aqueous pocket around the 7-methoxy-substituent is generated by replacement of mammalian residues Y224 and K227 with M219 and T222 in *T.* *gondii*, respectively. Moreover, a T222P substitution resulted in resistance to 7-methoxy ELQs^36^. To test this hypothesis, we synthesized and tested ELQs with a longer 7-ethoxy substituent in combination with chlorine (ELQ-804), fluorine (ELQ-807) and hydrogen (ELQ-627) at position 6 (Fig. 5d,f and Extended Data Table 1). As predicted, the extended 7-ethoxy substituent increased potency compared with the analogous 7-methoxy-substituent. ELQ-804, ELQ-807 and ELQ-627 were fourfold, threefold and tenfold more potent than their respective 7-methoxy analogs. We propose that the most potent compound, ELQ-627, possesses the greatest potential for extending into the pocket owing to the smallest 6-position substituent, hydrogen, paired with the 7-position ethoxy group. The EC_50_ values of each 7-methoxy compound compared with its 7-ethoxy analog was statistically different (*P* < 0.0001). The EC_50_ of ELQ-627 was 0.12 nM, which was 27-fold more potent than ELQ-316, a lead preclinical candidate for toxoplasmosis that is highly effective and well-tolerated in mouse models of acute and latent toxoplasmosis compared with clinically used drugs and advanced preclinical compounds^42^. The discovery and validation of a unique binding pocket in the *T.* *gondii* Cyt-b Q_i_ site provides a readily exploitable feature of the apicomplexan target for structure-guided drug design. The cryo-EM structure of the apicomplexan CIII will advance the development of pathogen-specific, highly potent Cyt-b inhibitors to treat devastating apicomplexan diseases.

## Methods

### Parasite cell culture and genetic manipulation

*T.* *gondii* tachyzoites were cultured in HFF, sourced from American Type Culture Collection (SCRC-1041). HFFs and parasites were cultured in Dulbecco’s modified Eagle medium, containing 4.5 g l^−1^ glucose, supplemented with 10% (v/v) fetal bovine serum (FBS), 4 mM L-glutamine and penicillin or streptomycin antibiotics and grown at 37 °C with 5% CO_2_.

To generate the Rieske-3xFLAG line (C-terminal triple FLAG epitope tagging), a gRNA targeting the stop codon of TGGT1_320220 was identified using ChopChop^44^ and cloned into a U6 promoter and Cas9–green fluorescent protein expressing vector (Tub-Cas9YFP-pU6-ccdB-tracrRNA)^45^ using the BsaI restriction site. The CAT selection cassette and triple FLAG epitope were amplified by PCR from a pLIC.TEV.3xFLAG.CATΔpacI plasmid^46^. The gRNA–CAS9 vector–PCR product mixture was transfected into the TATi∆ku80 (ref. ^47^) line by electroporation and cassette integration was selected with chloramphenicol. Positive clones were isolated by serial dilution and confirmed by PCR analysis.

For the ApiCox knockout line, a gRNA targeting the stop codon of TGGT1_316255 was identified via ChopChop^44^ and cloned as above into Tub-Cas9YFP-pU6-ccdB-tracrRNA. A dihydrofolate reductase (DHFR) resistance cassette was amplified using the pDT7S4 plasmid as template^47^ and using primers containing 50 bp of sequence homology to regions upstream and downstream of the TGGT1_316255 open reading frame. The gRNA–Cas9 vector–PCR product mixture was transfected into the either a Cox2a-HA, QCR2-HA^8^ or mNEON::Δku80 (ref. ^48^) parental line by electroporation and cassette integration was selected with pyrimethamine. Positive clones were isolated by serial dilution and confirmed by PCR analysis. For the complementation of the knockout cell line, cDNA was cloned into a pTUB8mycGFPMyoATy expression vector via EcoRI and NsiI restriction sites^49^, plasmid electroporated and selected with mycophenolic acid (25 mg ml^−1^) and xanthine (50 mg ml^−1^).

### Growth analysis

Replication assay: parental or ApiCox10-KO parasites were inoculated into HFF cells grown on a glass coverslip and left to invade and replicate for 24 h. Parasites were detected via immunofluorescence using the GAP45 antibody [1:1,000]^50^. The number of vacuoles containing 1, 2, 4 or 8+ parasites were counted for more than 250 vacuoles per replicate. Four independent experiments were performed.

Competition assay: parental or ApiCox10-KO parasites in the mNEON::Δku80 background^48^ were counted, resuspended in fluorescence-activated cell sorting (FACS) buffer (25 mM HEPES, 5 mM EDTA and 1% w/v FBS) and mixed in a ≈1:1 ratio with wild-type tdTomato::Δku80 parasites and the mixed population inoculated in cell culture. These mixed populations were passaged six times. At each passage a portion of the population was analyzed using BD FACS Celesta (BD Biosciences). Data were analyzed using the FlowJo v10.8.1 software (BD biosciences).

Growth inhibition for ELQ EC_50_: *T.* *gondii* proliferation inhibition with ELQs was measured in a 96-well assay using an RH *T.* *gondii* strain expressing beta-galactosidase cultured in HFF. Compounds dissolved in dimethylsulfoxide (DMSO) were diluted serially across the plate in four replicate rows by fourfold dilutions with a control column receiving no compounds. Then, 4,000 *T.* *gondii* tachyzoites were added to each well. After 3 days of incubation (37 °C, 5% CO_2_), the media were replaced with a solution of chlorophenol red-β-d-galactopyranoside and NP-40. The absorbance of each well was measured at 575 nm in a Molecular Devices SpectraMax 190 plate reader. Each compound was tested in at least three independent experiments. Absorbance was plotted against the base-10log of compound concentration and fitted to a four-parameter model of the Hill equation to estimate the EC_50_ for each compound. The 95% CIs of the mean, two-tailed unpaired *t*-tests and EC_50_ values were calculated using GraphPad Prism v8.4.3 software.

### Blue native and SDS–PAGE and immunoblot analysis

For native polyacrylamide gel electrophoresis (PAGE) analysis, parasites were filtered through a 3.0-µm polycarbonate filter, washed with ice cold phosphate buffered saline (PBS) and resuspended in native PAGE sample buffer (Thermo Fisher) supplemented with 1% n-dodecyl β-D-maltoside (β-DDM) or 1% digitonin. After 30 min incubation at 4 °C, samples were centrifuged at 18,000*g* for 30 min at 4 °C and the supernatant mixed with Coomassie G250 to a final concentration of 0.25% w/v. Samples were separated on a native PAGE 4–16% (for monomer detection) or a 3–12% (for supercomplex detection) Bis-Tris gel. NativeMark and a bovine mitochondrial membrane preparation was used as a molecular weight marker. SDS–PAGE, immunoblot analysis and DAB staining were performed as described previously^8,46^. The following primary antibodies were used: anti-HA (Roche, clone 3F10) (1:500), anti-TOM40 (1:2,000)^51^, anti-MYS (1:2,000)^52^, anti-Ty^53^ (1:800), anti-CDPK1 (1:10,000)^54^ and anti-FLAG (Thermo Fisher, clone FG4R) (1:2,000). SDS–PAGE immunoblots were then labeled with secondary fluorescent antibodies (LI-COR: anti-mouse IRDye 800CW, anti-rabbit IRDye 680RD and anti-rat IRDye 800CW, all 1:10,000) and imaged with an Odyssey CLx. Native PAGE immunoblots were labeled with secondary horseradish peroxidase-conjugated antibodies (anti-rat, immunoglobulin G H&L horseradish peroxidase, abcam, 1:5,000; anti-rabbit, immunoglobulin G H&L horseradish peroxidase conjugate, Promega, 1:10,000) and chemiluminescence detection using Pierce ECL western blotting substrate and either an X-ray film or an iBright FL1000 imager (Invitrogen).

### Analysis of respiratory rate

OCR and extracellular acidification rate (ECAR) were measured using a Seahorse XF HS Mini Analyzer v3.0.0.41 (Agilent Technologies) as described previously^55^. Each of the six independent experiments was performed with parental and ApiCox10-KO parasites with two technical replicates.

### Flow cytometry using JC-1

Parasites were filtered through a 3.0 µm polycarbonate filter and incubated with 1.5 µM JC-1 (5,5′,6,6′-tetrachloro-1,1′,3,3′-tetraethylbenzimidazolocarbocyanine iodide, Thermo Fisher Scientific, stock 1.5 mM in DMSO) for 15 min at 37 °C. Treatment with 10 μM valinomycin was used as a depolarizing control. Cells were pelleted and resuspended in 1–2 ml FACS buffer (25 mM HEPES, 5 mM EDTA and 1% w/v FBS) before analysis using a BD FACS Celesta analyzer and data acquisition using FACSDiva software v9 (BD Biosciences). Unstained controls were used to define gates for analysis. In total, 50,000 events per treatment were collected, and data were analyzed using FlowJo v10.8.1 software (BD Biosciences).

### Immunofluorescence assay

Parasites were inoculated on fresh HFFs on glass coverslips. After 1 day, cells were fixed with 4% paraformaldehyde. Cells were permeabilized and blocked with a solution of 2% bovine serum albumin and 0.2% triton X-100 in PBS before incubation with primary antibodies (anti-Ty^53^, anti-MYS^52^ and anti-TOM40 (ref. ^51^)), 1:1,000, followed by secondary antibodies (Alexa Fluor Goat anti-Mouse 488 Invitrogen A-11001, 1:1,000 and Alexa Fluor Goat anti-Rabbit 594 Invitrogen A-11012, 1:1,000). Coverslips were mounted on slides with Fluoromount-G mounting media containing 4′,6-diamidino-2-phenylindole (Southern Biotech, 0100–20). Slides were visualized on a DeltaVision Core microscope (Applied Precision) using the 100× objective and *z*-stacking. Images were deconvolved using SoftWoRx v5.5 software and processed using FIJI software v1.5.2 (ref. ^56^).

### Transmission electron microscopy for mitochondrial cristae analysis

Parental and ApiCox10-KO parasites were allowed to invade HFF and form vacuoles. Cells were then fixed with fixation buffer (2.5% (v/v) glutaraldehyde, 4% (w/v) paraformaldehyde, in 0.1 M cacodylate buffer, pH 7.2), washed in 0.1 M cacodylate buffer, pH 7.2 and post-fixed in 1% (w/v) OsO_4_, 1.25% (w/v) K_4_[Fe(CN)_6_] for 1 h on ice. After several washes in the same buffer, the samples were en bloc stained with 0.5% (w/v) uranyl acetate in water for 30 min. Thereafter, samples were washed with water, dehydrated in ascending acetone series and resin embedded. Ultrathin sections (~50 nm thick) were collected and imaged on a JEOL 1200 Transmission electron microscope (JEOL) operated at 80 kV. Images were analyzed in FIJI software and the number of cristae per unit mitochondrial area was calculated. Cristae density: parental: 62.57 ± 19.19 cristae µm^−2^, mean, s.d., *n* = 100. ApiCox10-KO: 58.55 ± 17.97 cristae µm^−2^; mean, s.d., *n* = 100. Mitochondrial area: parental: 0.09739 ± 0.09649 µm^2^, mean, s.d. *n* = 100. ApiCox10-KO: 0.09441 ± 0.06876 µm^2^, mean, s.d., *n* = 100.

### MitoSOX staining

MitoSOX staining to assess oxidative stress in the mitochondria was performed as described previously^48^ with minor modifications. Briefly, parasites were grown in the presence or absence of 2 mM ferric ammonium chloride, shown to increase mitochondrial oxidative stress, for 6 h, before parasites were filtered through a 3.0 µm polycarbonate filter and incubated with 1 µM MitoSOX (Thermo Fisher, M36008) for 30 min at 37 °C. Cells were pelleted and resuspended in 2 ml FACS buffer (25 mM HEPES, 5 mM EDTA and 1% w/v FBS) before analysis using BD FACS Celesta analyzer and data acquisition using FACSDiva software v9 (BD Biosciences). Parasites were gated on forward and side scatter and on green fluorescence before the red fluorescent signal was analyzed. In total, 100,000 events per treatment were collected and data were analyzed using the FlowJo v10.8.1 software (BD biosciences).

### Immunoprecipitation and mass spectrometry

Immunoprecipitations for identification by mass spectrometry were performed as described previously^55^ with minor modifications. Cox2a-HA and ApiCox10-KO/Cox2a-HA parasites were lysed in a lysis buffer containing 1% digitonin. Elutions containing equal amount of protein were sent for mass spectrometry analysis. Four independent experiments were performed. Proteins detected in at least three out of the four experiments are displayed in a volcano plot. Data analysis to generate a volcano plot was performed using Perseus v1.6.12.0; samples were compared using a two-sided *t*-test, the false discovery rate was set to 0.01 and the significance threshold was set to 2.

### Affinity purification of the dimeric *T. gondii* CIII

Immunoprecipitation of Rieske-TEV-3xFLAG parasites was performed using anti-FLAG M2 affinity agarose gel (Merck). Parasites (≈1 × 10^10^) were incubated in 4 ml buffer (150 mM NaCl, 2 mM EDTA, 50 mM Tris–HCl pH 7.4) containing 2% β-DDM for 2 h at 4 °C before centrifugation at 18,000*g* for 30 min at 4 °C. The supernatant was incubated with FLAG affinity gel overnight at 4 °C. Then the affinity gel was washed three times with buffer containing 0.05% β-DDM before elution with a FLAG peptide solution (150 µg ml^−1^) for 1 h at 4 °C. The eluate was concentrated to ≈50 µl in a vivaspin 500 filter (100 kDa molecular weight cutoff) and subjected to gel filtration on a Superose 6 Increase 3.2/300 column (GE Healthcare) in 150 mM NaCl, 2 mM EDTA, 50 mM Tris–HCl pH 7.4 and 0.05% β-DDM to separate it from aggregates and FLAG peptide. CIII-containing fractions were pooled and incubated with amphipol A8-35 (Anatrace) in a molar ratio of 1:5 for 4 h at 4 °C, followed by the addition of Bio-Beads SM-2 Resin (Bio-Rad) in a molar ratio of 1:20 for 16 h at 4 °C to remove detergent. The sample was concentrated and gel filtrated as before, followed by a final spin-column concentration to 0.94 mg ml^−1^. ELQ-300 (in DMSO) was added to a final concentration of 10 µM before sample vitrification.

### Mitochondrial isolation

Parasite culturing and mitochondria purification was performed as previously described^13^. Briefly, *T.* *gondii* RH tachyzoites were grown in Vero cells in Dulbecco’s modified Eagle medium supplemented with 10% (v/v) FBS, 4 mM of l-glutamine and penicillin or streptomycin antibiotics at 37 °C with 5% (v/v) CO_2_. For each mitochondrial preparation ≈100 T150 flasks were collected at >80% host-cell lysis and passed through 23G needles to fully lyse any remaining host cells. Parasites were pelleted by centrifugation at 1,500*g* for 10 min at 4 °C, washed in PBS and then resuspended in buffer containing 210 mM of mannitol, 70 mM of sucrose, 50 mM of HEPES–KOH pH 7.4, 1 mM of EGTA, 5 mM of EDTA, 10 mM of KCl and 1 mM of dithiothreitol (DTT) to 5 × 10^8^ cells ml^−1^. Parasites were lysed by successive rounds of nitrogen cavitation (2,500 PSI, 15 min incubation on ice) until >95% lysis (confirmed by light microscopy). After each round, the lysate was centrifuged at 1,500*g* for 15 min at 4 °C, the supernatant was collected and the pellet resuspended in the same volume for further lysis. The final combined lysate was centrifuged as before to remove unbroken cells and the supernatant was centrifuged at 16,000*g* for 30 min at 4 °C. The resulting crude mitochondrial pellet was further purified on a discontinuous sucrose gradient in 20 mM of HEPES–KOH pH 7.4, 2 mM of EDTA, 15%/23%/32%/60% (w/v) sucrose by centrifugation (103,745*g*, 1 h, 4 °C) in an SW41 rotor (Beckman Coulter) and enriched mitochondria were collected from the 32–60% (w/v) interface.

### Purification of respiratory supercomplexes

Enriched mitochondria were lysed in a total volume of 34 ml buffer containing 25 mM HEPES–KOH pH 7.5, 25 mM KCl, 15 mM MgOAc_2_, 2% (w/v) digitonin, 2 mM DTT and one tablet of EDTA-free protease inhibitor cocktail for 2 h at 4 °C and the lysate was cleared by centrifugation at 30,000*g* for 20 min at 4 °C. The supernatant was layered on a sucrose cushion in buffer of 1 M sucrose, 25 mM HEPES–KOH pH 7.5, 25 mM KCl, 15 mM MgOAc_2_, 2% digitonin and 2 mM DTT, and centrifuged at 230,759*g* for 4 h at 4 °C in a Ti70 rotor (Beckman Coulter). The resulting pellet was resuspended in 200 µl 25 mM HEPES–KOH pH 7.5, 25 mM KCl, 15 mM MgOAc_2_, 2 mM DTT and 0.1% digitonin, and gel filtrated over a Superose 6 Increase 3.2/300 column (GE Healthcare). Fractions corresponding to the respiratory chain supercomplex were pooled and concentrated to 25 µl in a vivaspin 500 filter (100 kDa molecular weight cutoff). Atovaquone and ELQ-300 (in DMSO) were added in a 100-fold dilution to a final concentration of 10 µM before sample vitrification.

### Electron cryo-microscopy and data processing

Next, 3 µl of sample (~0.2–0.5 mg ml^−1^) were applied to glow-discharged Quantifoil R2/2 Cu grids with a 2-nm-thin carbon support layer and vitrified by plunge-freezing into liquid ethane after blotting for 3 s. Cryo-EM was performed on a Titan Krios operated at 300 kV at a magnification of 165 kx (0.83 Å per pixel) with a K3 quantum camera (slit width 20 eV) at an exposure rate of 17 electrons pixel^−1^ s^−1^ with a 1.75 s exposure fractionated into 40 frames using EPU 1.12 software (Thermo Fisher Scientific).

Initial rounds of 2D classification were performed to generate classes for reference-based particle picking, which was performed in RELION4, resulting in 2,658,324 picked particles. To address sample heterogeneity, the resulting classes of a subsequent 2D classification (cryoSPARC4.6) were manually grouped and subjected to ab initio model generation (cryoSPARC), which resulted in six different 3D references, corresponding to the *T.* *gondii* ATP synthase dimer and supercomplex, the *C.* *sabaeus* ATP synthase and I–III_2_–IV supercomplex, and mitochondrial large ribosomal subunit, as well as an unidentified C_7_-symmetric complex (Supplementary Fig. 1d). These six maps were used as input to heterogeneous refinements, resulting in the particle distribution shown in Supplementary Fig. 1, with 432 k and 637 k particles for *T.* *gondii* and *C.* *sabaeus*, respectively. Subsequent masked refinements of the *T.* *gondii* supercomplex yielded maps of the CIII and CIV subcomplexes at 2.79 and 3.16 Å, respectively.

Data collection of the ELQ-300-inhibited *T.* *gondii* complex-III dataset was performed on a Titan Krios, as described above, resulting in 46,370 movies. Image processing was performed in cryoSPARC. Templates for particle picking were generated by initial rounds of 2D classification. 12,822,652 particles were picked and cleaned by 2D classification resulting in 4,451,128 particles. To reduce map anisotropy and increase interpretability, the number of top views was reduced, resulting in a final number of 2,056,878 particles. Non-uniform refinement resulted in a 1.9 Å structure which was improved to 1.83 Å by cryo-EM density modification in PHENIX 1.21 (ref. ^57^).

All final maps were generated from contrast transfer function (CTF)-refined particles. All resolution estimates are according to Fourier shell correlations (FSC) that were calculated from independently refined half-maps using the 0.143 criterion with correction for the effect of the applied masks (Extended Data Fig. 1).

### Atomic model building, homology modeling and analysis

An initial atomic model of the *T.* *gondii* III_2_–IV supercomplex was assembled by fitting the known conserved subunits, as predicted by AlphaFold2 (ref. ^58^) into the cryo-EM maps. Newly identified subunits were built de novo and identified by BLAST searches following manual modeling in *Coot* 0.96 (ref. ^59^). At the modeled N-terminus of the *T.* *gondii* Cyt-b, we identified cryo-EM density preceding residue Met10, which is consistent with a Phe9. While this observation is not explained by the mRNA sequence, it is consistent with the high-resolution cryo-EM map.

For atomic model building of the *C.* *sabaeus* CIII, a homology model of the murine CIII (Protein Data Bank (PDB) 7o37) was generated using the SWISS-MODEL webserver, followed by rebuilding and inhibitor fitting in Coot and structure refinement in PHENIX 1.19. Homology modeling of the *P.* *falciparum* QCR6 homolog was performed in SWISS-MODEL. For the mapping of Cyt-*c* binding sites, PDB 3CX5 (ref. ^60^) and 5IY5 (ref. ^61^) were fitted to the *T.* *gondii* complexes III_2_ and IV, respectively, to reveal cytochrome binding sites.

### Data visualization and multiple sequence alignment

Prediction of membrane positions was performed using the OREMPRO webserver^62^. Structure-based multiple sequence alignment of QCR6 was performed in Chimera-1.14 (ref. ^63^). Images of the structures were generated with ChimeraX 1.6.1 (ref. ^64^).

#### Subunit annotation and nomenclature

For CIII subunits, TGGT1_214250, previously named apicomplexan-specific QCR11 (ref. ^8^), was reassigned as a homolog of conserved subunit QCR10 and thus renamed TgQCR10. The newly identified TGGT1_312940 was named TgQCR13 to avoid changing the already named TgQCR12. For CIV subunits, we followed a previously established ‘ApiCox’ nomenclature^11^. However, structural similarity showed that previously named ApicCox23, 25, 35 and 14 correspond to conserved subunits Cox4, 6A, 6C and 7a, respectively. We also reassigned TGGT1_306670 as NDUFA4. Furthermore, we newly identified the conserved subunit Cox2c and Cox7c directly from the cryo-EM density. We also reassigned the previously detected subunit TGGT1_257160 as conserved Cox8a and, following the established nomenclature, novel clade-conserved subunits ApiCox22, 20, 5, 10 and 7.

### Reporting summary

Further information on research design is available in the Nature Portfolio Reporting Summary linked to this article.

## Online content

Any methods, additional references, Nature Portfolio reporting summaries, source data, extended data, supplementary information, acknowledgments, peer review information; details of author contributions and competing interests; and statements of data and code availability are available at 10.1038/s41594-025-01531-7.

## Supplementary information

Supplementary Information Supplementary Methods, Discussion, Notes and References.

Reporting Summary

Supplementary Data 1 Mass spectometry data from Cox2a-HA and ApiCox10-KO/Cox2a-HA immunoprecipitations. log_2_FC and log_10_ *P*-value data used to generate Extended Data Fig. 7h. Complex III and IV subunits detected.

Supplementary Table 1 *T. gondii* supercomplex subunits and *C. sabaeus* complex III subunits.

Supplementary Table 2 Table of primer sequences

Supplementary Video 1 Supplementary Video 1.

## Source data

Source Data Fig. 3 Uncropped gel images (pdf) and original data values (xlsx).

Source Data Extended Data Fig. 2/Table 2 Subunit homology search E values and hydrophobicity calculations.

Source Data Extended Data Fig. 4/Table 4 Uniprot entries.

Source Data Extended Data Fig. 6/Table 6 Raw blots and gels (pdf) and raw images and data values (xlsx).

Source Data Extended Data Fig. 9/Table 9 Uncropped blots and gels.

## Figures and Tables

**Extended Data Table 1:**
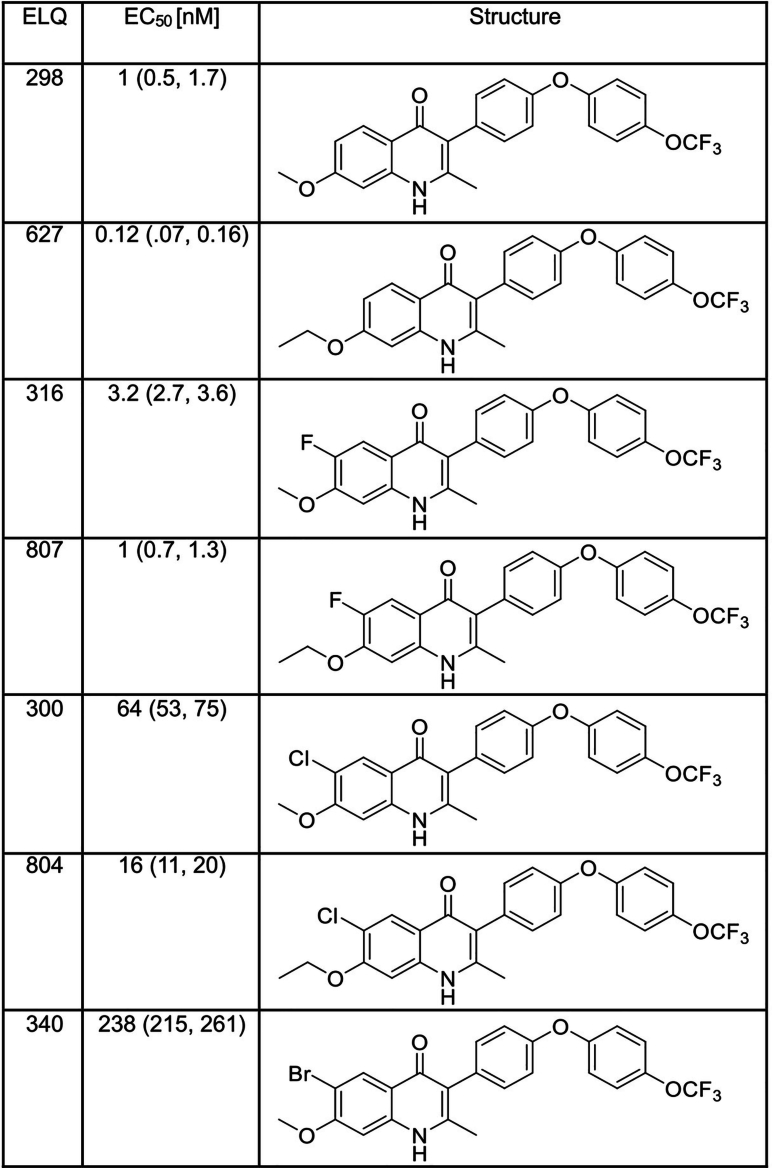
Endochin-like Quinolone (ELQ) inhibition of *Toxoplasma gondii* proliferation

50% Effective concentrations (EC_50_) with 95% confidence intervals (95% confidence interval) for ELQs against *Toxoplasma gondii*.

## Data Availability

The atomic coordinates were deposited in the PDB under accession numbers 9I4X (*T.* *gondii* complex III with ELQ-300/ATQ), 9I4Y (*T.* *gondii* CIV), 9H8T (*C.* *sabaeus* CIII with ATQ), and 9G9T (*T.* *gondii* CIII with ELQ-300). The cryo-EM maps have been deposited in the Electron Microscopy Data Bank (EMDB) under the respective accession numbers: EMD-52621 (*T.* *gondii* complex III with ELQ-300/ATQ), EMD-52622 (*T.* *gondii* CIV), EMD-51939 (*C.* *sabaeus* CIII with ATQ), and EMD-51157 (*T.* *gondii* CIII with ELQ-300). The atomic coordinates that were used in this study are: PDB 7O3C (murine III_2_–IV supercomplex), 5IY5 (cytochrome *c*) and 3CX5 (complex III with bound cytochrome *c*). Full versions of all gels are provided in the source file. The mass spectrometry proteomics data have been deposited to the ProteomeXchange Consortium via the PRIDE partner repository with the dataset identifier PXD053932. Supplementary Information is available. It includes Supplementary Methods (parasite cell culture and genetic manipulation; mitochondrial isolation; purification of respiratory supercomplexes; electron cryo-microscopy and data processing; atomic model building, homology modeling and analysis; data visualization and multiple sequence alignment; growth analysis; blue native (BN) and SDS–PAGE and immunoblot analysis; analysis of respiratory rate; compound synthesis); Supplementary Tables 1 and 2, Supplementary Discussion and Supplementary Notes. Source data are provided with this paper.
