# Author Correction: Structure, assembly and inhibition of the *Toxoplasma gondii* respiratory chain supercomplex

**DOI:** 10.1038/s41594-026-01824-5

**Published:** 2026-05-22

**Authors:** Andrew E. MacLean, Shikha Shikha, Mariana Ferreira Silva, Max J. Gramelspacher, Aaron Nilsen, Katherine M. Liebman, Sovitj Pou, Rolf W. Winter, Amit Meir, Michael K. Riscoe, J. Stone Doggett, Lilach Sheiner, Alexander Mühleip

**Affiliations:** 1 School of Infection and Immunity, University of Glasgow, Glasgow, UK; 2 Glasgow Centre for Parasitology, University of Glasgow, Glasgow, UK; 3 VA Portland Health Care System, Portland, OR USA; 4 Medicinal Chemistry Core, Oregon Health and Science University, Portland, OR USA; 5 Centre for Virus Research, University of Glasgow, Glasgow, UK; 6 Department of Microbiology and Molecular Immunology, Oregon Health and Science University, Portland, OR USA; 7 School of Medicine Division of Infectious Diseases, Oregon Health and Science University, Portland, OR USA; 8 Institute of Biotechnology, Helsinki Institute of Life Science HiLIFE, University of Helsinki, Helsinki, Finland

**Keywords:** Mitochondrial proteins, Drug discovery, Cryoelectron tomography

Correction to: *Nature Structural & Molecular Biology* 10.1038/s41594-025-01531-7, published online 19 May 2025.

In the version of this article initially published, the competing interests section stated that the authors declare no competing interests and has now been amended to “Oregon Health and Science University, M.K.R., J.S.D. and A.N. have a financial interest in the ELQ compounds which were used in this research, and which have been licensed for commercial development. This potential conflict of interest has been reviewed and managed by Oregon Health and Science University. The other authors declare no competing interests” in the HTML and PDF versions of the article.

