# Supplementary material for: Structure, assembly and inhibition of the Toxoplasma gondii respiratory chain supercomplex
Source: Nat Struct Mol Biol. 2025 May 19;32(8):1424–33. doi: 10.1038/s41594-025-01531-7 (PMC12350165; doi:10.1038/s41594-025-01531-7)
Supplement: Supplementary file 1 — Supplementary Methods, Discussion, Notes and References. [file 41594_2025_1531_MOESM1_ESM.pdf]

nature structural & molecular biology

Supplementary information

https://doi.org/10.1038/s41594-025-01531-7

Structure, assembly and inhibition of the *Toxoplasma gondii* respiratory chain supercomplex

In the format provided by the authors and unedited

Supplementary Information:
Supplementary Methods

Compound synthesis

Unless otherwise stated all chemicals and reagents were from Sigma-Aldrich Chemical Company in St. Louis, MO (USA), Combi-Blocks in San Diego (CA), Fisher Scientific in Waltham (MA), or TCI America, Portland (OR) and were used as received. Melting points were obtained in an Optimelt Automated Melting point system from Stanford Research Systems, Sunnyvale, CA (USA). ^{1}H, ^{13}C, and ^{19}F NMR spectra were obtained using a Bruker AMX-400 MHz NMR spectrometer. Chemical shifts are reported in parts per million (ppm), relative to internal (TMS) peak for ^{1}H and ^{13}C. The NMR raw data were analyzed using iNMR Spectrum Analyst software. HPLC analyses were performed using an Agilent 1260 Infinity instrument with detection at 254 nm, using a Phenomenex Luna® 5μm C8(2) 100 Å reverse phase LC 150 x 4.6 mm column at 40°C, eluting with a gradient of 25%:75% to 10%:80% v:v A:B, where A was 0.05% formic acid in milliQ water and B was 0.05% formic acid in methanol. High resolution mass spectrometry (HRMS) was performed at the Portland State University Bioanalytical Mass Spectrometry Facility using a Vanquish UHPLC/HPLC system coupled with a high-resolution (345000) Q Exactive Orbitrap mass spectrometer. ELQ-300, ELQ-316, and ELQ-298 were synthesized by methods previously described[1,2]. Using analogous methods, ELQ-627, ELQ-804, and ELQ-807 were synthesized as follows.

<u>**ELQ-627** (7-ethoxy-2-methyl-3-(4-(4-(trifluoromethoxy)phenoxy)phenyl)quinolin-4(1H)-one):</u>

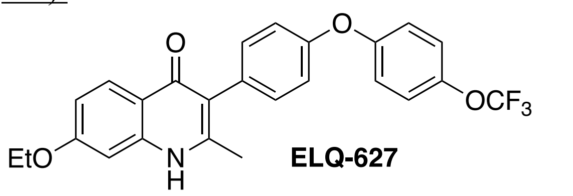

ELQ-627

Part 1: Preparation of the Schiff base (Ethyl (Z)-3-((3-ethoxyphenyl)imino)-2-(4-(4-(trifluoromethoxy)phenoxy)phenyl)butanoate):

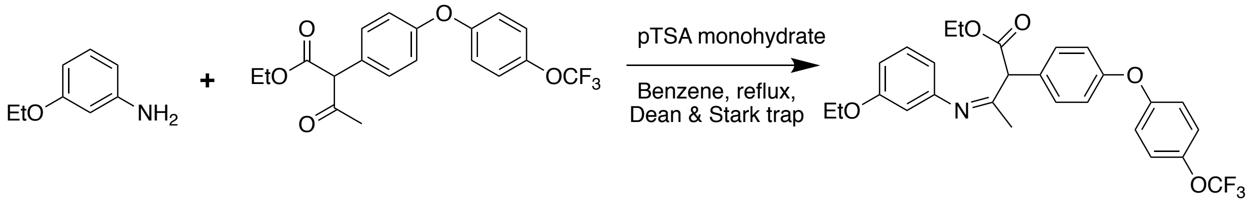

meta-Phenetidine (1.92 g, 0.014 mol) and ethyl 3-oxo-2-(4-(4-(trifluoromethoxy)phenoxy)phenyl)butanoate[2] containing 10 mole % of para-toluenesulfonic acid (5.60 g, thus 5.34 g, 0.014 mol, 1.0 eq of ester and 0.26 g, 0.0014 mol, 0.1 eq of para-toluenesulfonic acid) were heated at reflux in 70 mL of benzene for 2 days in a flask equipped with a Dean and Stark water separator. Removal of the solvent under reduced pressure with warming afforded the crude Schiff base as a brown oil. This was used without analysis or purification in the ensuing reaction.

*Part 2: Formation of **ELQ-627** (7-ethoxy-2-methyl-3-(4-(4-(trifluoromethoxy)phenoxy)phenyl)quinolin-4(1H)-one) by Conrad-Limpach cyclization:*

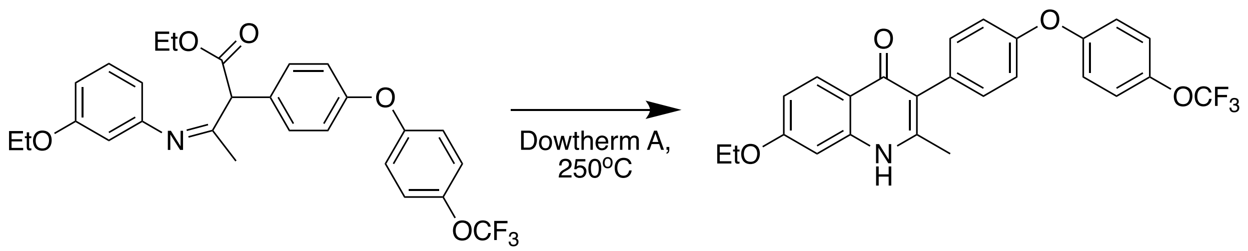

The crude Schiff base (ethyl (Z)-3-((3-ethoxyphenyl)imino)-2-(4-(4-(trifluoromethoxy)phenoxy)phenyl)butanoate) was taken up in 5 mL hot Dowtherm A and added over 10 minutes to 65 mL boiling Dowtherm A (250°C), together with a further 5 mL hot Dowtherm A used to rinse the flask. After 2 minutes' further heating, the reaction was removed from the heat and stirred while cooling to room temperature. The reaction was then diluted with 300 mL hexanes. After brief stirring, the precipitate was recovered by vacuum filtration, rinsing with hexanes followed by 30 mL ethyl acetate. The resulting beige solid (2.41 g) was recrystallized from DMF (20 mL) to give fine, white crystals (0.89 g, 14% over two steps from *m*-phenetidine, mp = 275.3-277.3°C (dec.), ^{1}H-NMR (400 MHz; DMSO-d$_6$): δ 11.44 (s, 1H), 7.97 (d, *J* = 8.7 Hz, 1H), 7.44-7.39 (m, 2H), 7.29-7.26 (m, 2H), 7.18-7.14 (m, 2H), 7.08-7.05 (m, 2H), 6.90-6.86 (m, 2H), 4.12 (q, *J* = 7.0 Hz, 2H), 2.23 (s, 3H), 1.40 (t, *J* = 7.0 Hz, 3H).); ^{19}F NMR (376 MHz; DMSO): δ -57.2 (s); ^{13}C NMR (101 MHz; DMSO): δ 175.0, 161.4, 156.3, 155.0, 146.6, 141.5, 133.3, 132.4, 127.7, 123.5, 120.17, 120.09, 119.0, 118.7, 113.5, 99.5, 63.9, 19.3, 15.0; HRMS calculated for $C_{25}H_{21}F_3NO_4$ $[M + H]^+$ = 456.1417, observed $[M + H]^+$ = 456.1409. HPLC analysis indicated that the product was 99.33% pure.

ELQ-804 *((ethyl (Z)-3-((3-ethoxy-4-chlorophenyl)imino)-2-(4-(4-(trifluoromethoxy)phenoxy)phenyl)butanoate):*

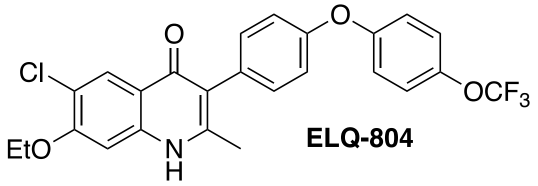

*Part 1: Preparation of the Schiff base (*ethyl (*Z*)-3-((3-ethoxy-4-chlorophenyl)imino)-2-(4-(4-(trifluoromethoxy)phenoxy)phenyl)butanoate*):*

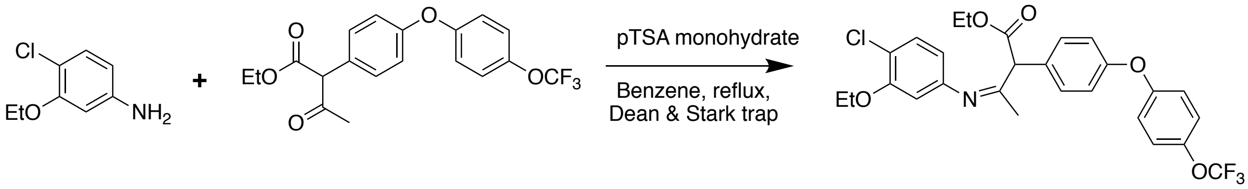

3-Ethoxy-4-chloroaniline (0.1.5 g, 0.0087 mol) and ethyl 3-oxo-2-(4-(4-(trifluoromethoxy)phenoxy)phenyl)butanoate[2,3] containing 10 mole % of para-toluenesulfonic acid (3.51 g, thus 3.35 g, 0.0087 mol, 1.0 eq of ester and 0.16 g, 0.00087 mol, 0.1 eq of *para*-toluenesulfonic acid) were heated at reflux in 70 mL of benzene for 24 hours in a flask equipped with a Dean and Stark water separator. Removal of the solvent under reduced pressure with warming afforded the crude Schiff base as a golden brown oil. This was used without analysis or purification in the ensuing reaction.

*Part 2: Formation of **ELQ-804** ((ethyl (Z)-3-((3-ethoxy-4-chlorophenyl)imino)-2-(4-(4-(trifluoromethoxy)phenoxy)phenyl)butanoate) via Conrad-Limpach cyclization:*

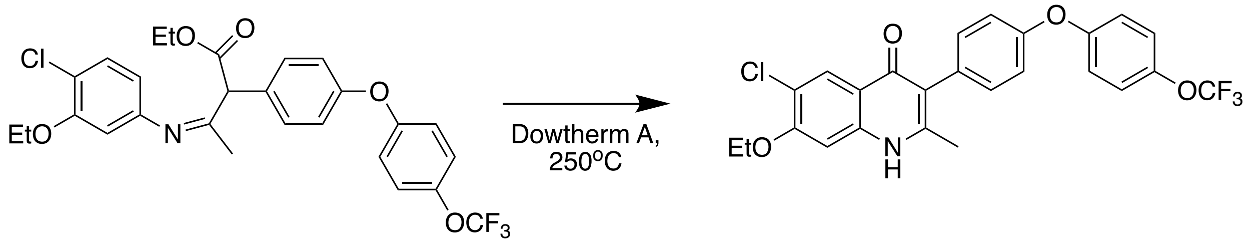

The crude Schiff base (ethyl (*Z*)-3-((3-ethoxy-4-chlorophenyl)imino)-2-(4-(4-(trifluoromethoxy)phenoxy)phenyl)butanoate) was taken up in 8 mL hot Dowtherm A and added over 5 minutes to 100 mL boiling Dowtherm A (250°C), together with a further 7 mL hot Dowtherm A used to rinse the flask. After 5 minutes' further heating, the reaction was removed from the heat and stirred until lukewarm. The reaction was then diluted with 400 mL hexanes and stirring was continued until room temperature had been reached. The resulting precipitate was recovered by vacuum filtration, rinsing with 100 mL hexanes followed by 20 mL ethyl acetate and finally, 3 x 3 mL acetone. This afforded a pale yellow solid (1.85 g) that was recrystallized from a mixture of ethyl acetate (20 mL) and N,N-dimethylformamide (8 mL) to afford the desired product as white needles (1.03 g, 24% over two steps from 3-ethoxy-4-chloroaniline, mp = 302.9-303.7°C (dec.), ^{1}H-NMR (400 MHz; DMSO-d$_6$): δ 11.62 (s, 1H), 8.00 (s, 1H), 7.43-7.40 (m, 2H), 7.30-7.26 (m, 2H), 7.18-7.14 (m, 2H), 7.08-7.05 (m, 3H), 4.20 (q, *J* = 7.0 Hz, 2H), 2.23 (s, 3H), 1.45 (t, *J* = 6.9 Hz, 3H)); ^{19}F NMR (376 MHz; DMSO): δ -57.2 (s); ^{13}C NMR (101 MHz; DMSO): δ 174.0, 156.42, 156.26, 155.2, 147.1, 144.1, 140.0, 133.2, 132.0, 126.6, 123.5, 120.33, 120.24, 119.1, 118.74, 118.62, 100.4, 65.2, 19.4, 14.8. HRMS calculated for $C_{25}H_{20}Cl_1F_3NO_4$ $[M + H]^+$ = 490.1027, observed $[M + H]^+$ = 490.1024. HPLC analysis indicated that the product was 98.25% pure).

<u>**ELQ-807** ((ethyl (Z)-3-((3-ethoxy-4-fluorophenyl)imino)-2-(4-(4-(trifluoromethoxy)phenoxy)phenyl)butanoate):</u>

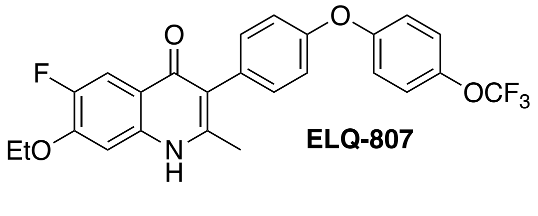

ELQ-807

*Part 1: Preparation of the Schiff base (*ethyl (*Z*)-3-((3-ethoxy-4-fluorophenyl)imino)-2-(4-(4-(trifluoromethoxy)phenoxy)phenyl)butanoate*):*

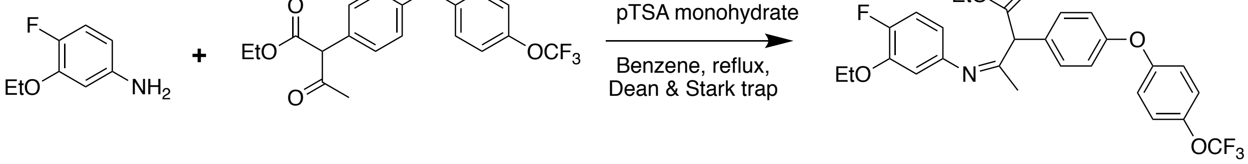

3-Ethoxy-4-fluoroaniline[3] (0.58 g, 0.0038 mol) and ethyl 3-oxo-2-(4-(4-(trifluoromethoxy)phenoxy)phenyl)butanoate[2] containing 10 mole % of para-toluenesulfonic acid (1.58 g, thus 1.51 g, 0.0039 mol, 1.05 eq of ester and 0.074 g, 0.00039 mol, 0.1 eq of *para*-toluenesulfonic acid) were heated at reflux in 80 mL of benzene for 3 days in a flask equipped with a Dean and Stark water separator. Removal of the solvent under reduced pressure with warming afforded the crude Schiff base as a rusty brown oil. This was used without analysis or purification in the ensuing reaction.

*Part 2: Formation of **ELQ-807** ((ethyl (Z)-3-((3-ethoxy-4-fluorophenyl)imino)-2-(4-(4-(trifluoromethoxy)phenoxy)phenyl)butanoate) via Conrad-Limpach cyclization:*

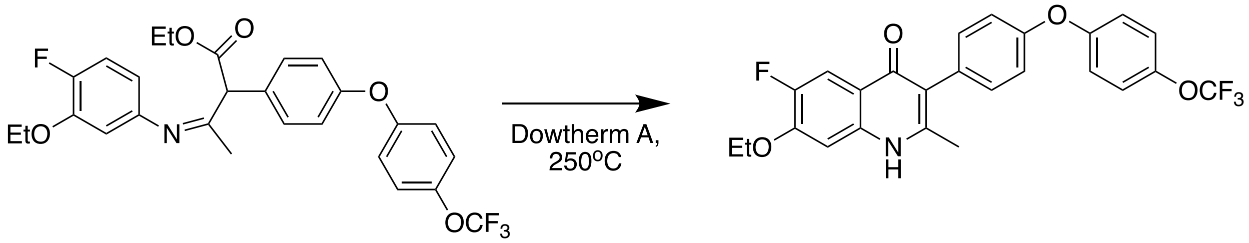

The crude Schiff base (ethyl (*Z*)-3-((3-ethoxy-4-fluorophenyl)imino)-2-(4-(4-(trifluoromethoxy)phenoxy)phenyl)butanoate) was taken up in 10 mL hot Dowtherm A and added over 6 minutes to 100 mL boiling Dowtherm A (250°C), together with a further 8 mL hot Dowtherm A used to rinse the flask. After 3 minutes' further heating, the reaction was removed from the heat and stirred while cooling to room temperature. The reaction was then diluted with 300 mL hexanes. After brief stirring, the precipitate was recovered by vacuum filtration, rinsing with 2x10 mL ethyl acetate followed by hexanes, then a further 3 x 8 mL ethyl acetate followed by 2 x 3 mL acetone. This afforded the desired product as a white solid (0.42 g, 23% over two steps from 3-ethoxy-4-fluoroaniline, mp = 285.0-286.6°C (dec.), ^{1}H-NMR (400 MHz; DMSO-d$_6$): δ 11.59 (s, 1H), 7.69 (d, J_F = 11.7 Hz, 1H), 7.44-7.40 (m, 2H), 7.29-7.26 (m, 2H), 7.18-7.14 (m, 2H), 7.09-7.05 (m, 3H), 4.20 (q, J = 7.0 Hz, 2H), 2.22-2.21 (m, 3H), 1.44 (t, J = 7.0 Hz, 3H); ^{19}F NMR (376 MHz; DMSO): δ -57.2 (s), -139.0 (dd, J_H = 11.86, 7.34); ^{13}C NMR (101 MHz; DMSO): δ 174.3, 156.3, 155.2, 150.6, 148.2, 146.7, 144.1, 137.5, 133.2, 132.1, 123.5, 120.2, 119.7, 118.7, 118.22, 118.17, 110.71, 110.52, 101.4, 65.0, 19.4, 14.8. HRMS calculated for $C_{25}H_{20}F_4NO_4$ $[M + H]^+$ = 474.1323, observed $[M + H]^+$ = 474.1324. HPLC analysis indicated that the product was 98.27% pure)

Supplementary Discussion

A second bound ELQ-300 molecule partially overlaps with the Q_o site

Within the Cyt-b cavity, atovaquone binds to the Q_o site, close to heme-b_L near the lumenal side of the membrane. In our TgCIII structure with ELQ-300, the second ELQ-300 molecule is bound within a matrix-side pocket of the same central cavity, surrounded by helices B,C,F and G (Fig. 5C) and located 7.8 Å away from heme b_H, from which it is separated by M97. While Y120 provides a hydrogen bond to the keto group of the quinolone, the remaining ligand interactions are predominantly hydrophobic, including a stacking interaction with W328. The binding sites of atovaquone and the second ELQ-300 molecule overlap partially (Fig. 5C), notably involving residues Y272, F289, I124, F269. By contrast, the part of the Q_o site accommodating the (naptho)-quinone headgroup is unoccupied, which is corroborated by the partial occupancy of the lumenal Rieske domain, indicating its mobility. Thus, our ELQ-300-inbhibited CIII structure shows that in addition to acting as a competitive inhibitor of the Q_i site, ELQ-300 can also enter the central quinone cavity and may interfere with accessibility of the Q_o site. Further studies are needed to determine the affinity of the second binding site and its potential contribution to the inhibitory profile of ELQ-300.

Atovaquone molecules bound in the Q_i and Q_o site of the mammalian complex III.

Whereas our *T. gondii* supercomplex structure in the presence of 10 µM each ELQ-300/atovaquone bound each ligand in the Q_i and Q_o sites, respectively, our *C. sabaeus* Cyt-b, was inhibited by atovaquone in both the Q_i and Q_o site. To establish hydrogen bonds in each site, atovaquone may adopt different tautomers. The fit of the atovaquone ligand in

the Q_o site is consistent with a hydrogen bond via an ionized 1-hydroxyl group that interacts with H239 of the Rieske subunit. By contrast, the atovaquone molecule in the Q_i site is likely bound as a different tautomer, with the 4-hydroxyl group interacting with D228. This would expose the carbonyl groups of C3 and C4 as hydrogen acceptors in a hydrogen bond formed with a protonated H201. Whereas previous studies have shown that the ionized binding mode of atovaquone in the Q_o site is independent of its protonation state in the bulk medium[4], our structure indicates that keto-enol tautomerism may enable the binding of atovaquone to both substrate binding sites of the mammalian Cyt-*b*.

Supplementary Notes

Statistics and reproducibility: Statistical analysis was performed in GraphPad Prism (v8.4.3). For the measurement of mitochondrial membrane potential using JC-1 dye, the mean ±s.d., from eight independent experiments is shown in Fig. 3G and p-values are from a one-way ANOVA followed by Tukey's correction for multiple pairwise comparisons. For the extracellular flux analysis measuring oxygen consumption, the graphs in Fig. 3H show mean ±s.d., from six independent experiments, p-value is from a two-tailed unpaired Student's t-test. For the growth competition in Fig. 3I the mean ±s.d. from four independent experiments is shown and the p-value calculated with a two-tailed one-sample t-test comparing values to passage 0. For the measurement of mitochondrial cristae density and mitochondrial area in Extended Data Fig. 6K,L the mean from 100 mitochondrial profiles is shown (±s.d. in K) and p-value generated from a two-tailed unpaired t-test. For the measurement of MitoSOX in Extended Data 6M the graphs show mean ± s.d., from 4 independent experiments and the p-value calculated using a one-way ANOVA followed by Turkey's multiple pairwise comparisons. For the quantification of parasites per vacuole in Extended Data Fig. 6, the graphs show mean ± s.d., from 4 independent experiments and the p-value calculated by multiple two-tailed t-tests with a Holm-Sidak correction applied. For the growth competition assay in Extended Data Fig. 6P points depict mean ± s.d., from 4 independent experiments and p-value determined from a one-way ANOVA, comparing abundance to passage 0, corrected for multiple comparisons (Dunnett) (ns, no significant difference; P3 $p = 0.0242$; P4 $p = 0.0022$; P5 $p = 0.0004$; P6 $p < 0.0001$).

All experiments involving native or SDS- PAGE to assess mETC formation (Figure 3 A-F, Extended Data Fig. 6I) were repeated at least three times independently with similar results. SDS-PAGE, DNA-PAGE and immunofluorescence experiments to confirm genetically modified lines (Extended Data Fig. 6B,C,E,F,G) were repeated at least two times independently with similar results. SDS-PAGE experiments to assess purification of Complex III (Extended Data Fig. 6 B-E) were repeated at least two times independently with similar results.

Videos

Supplementary Video 1: Atovaquone binds the *T. gondii* Q_o site via an induced fit.

Supplementary References

1 Nilsen, A. *et al.* Discovery, synthesis, and optimization of antimalarial 4(1H)-quinolone-3-diarylethers. *J Med Chem* **57**, 3818-3834 (2014). https://doi.org/10.1021/jm500147k

2 Pou, S. *et al.* A New Scalable Synthesis of ELQ-300, ELQ-316, and other Antiparasitic Quinolones. *Org Process Res Dev* **25**, 1841-1852 (2021). https://doi.org/10.1021/acs.oprd.1c00099

3 Zhang, Z. L., W.; Ruan, H.; Liu Y.; Mao F.;, Li, Y.; Zhou, Z;. NTCP inhibitors. (2019).

4 Birth, D., Kao, W. C. & Hunte, C. Structural analysis of atovaquone-inhibited cytochrome bc1 complex reveals the molecular basis of antimalarial drug action. *Nat Commun* **5**, 4029 (2014). https://doi.org/10.1038/ncomms5029
