# Supplementary material for: Structure, assembly and inhibition of the Toxoplasma gondii respiratory chain supercomplex
Source: Nat Struct Mol Biol. 2025 May 19;32(8):1424–33. doi: 10.1038/s41594-025-01531-7 (PMC12350165; doi:10.1038/s41594-025-01531-7)
Supplement: Supplementary file 2 — Reporting Summary [file 41594_2025_1531_MOESM2_ESM.pdf]

nature portfolio

Corresponding author(s): Lilach Sheiner
Alexander Mühleip

Last updated by author(s): Oct 22, 2024

Reporting Summary

Nature Portfolio wishes to improve the reproducibility of the work that we publish. This form provides structure for consistency and transparency in reporting. For further information on Nature Portfolio policies, see our Editorial Policies and the Editorial Policy Checklist.

Statistics

For all statistical analyses, confirm that the following items are present in the figure legend, table legend, main text, or Methods section.

n/a	Confirmed	
☐	☒	The exact sample size (*n*) for each experimental group/condition, given as a discrete number and unit of measurement
☐	☒	A statement on whether measurements were taken from distinct samples or whether the same sample was measured repeatedly
☐	☒	The statistical test(s) used AND whether they are one- or two-sided<br>*Only common tests should be described solely by name; describe more complex techniques in the Methods section.*
☒	☐	A description of all covariates tested
☐	☒	A description of any assumptions or corrections, such as tests of normality and adjustment for multiple comparisons
☐	☒	A full description of the statistical parameters including central tendency (e.g. means) or other basic estimates (e.g. regression coefficient) AND variation (e.g. standard deviation) or associated estimates of uncertainty (e.g. confidence intervals)
☐	☒	For null hypothesis testing, the test statistic (e.g. *F*, *t*, *r*) with confidence intervals, effect sizes, degrees of freedom and *P* value noted<br>*Give P values as exact values whenever suitable.*
☒	☐	For Bayesian analysis, information on the choice of priors and Markov chain Monte Carlo settings
☒	☐	For hierarchical and complex designs, identification of the appropriate level for tests and full reporting of outcomes
☒	☐	Estimates of effect sizes (e.g. Cohen's *d*, Pearson's *r*), indicating how they were calculated

Our web collection on statistics for biologists contains articles on many of the points above.

Software and code

Policy information about availability of computer code

Data collection	FACS: BD FACSDiva (v9)<br>Microscopy: SoftWoRx (v5.5)<br>Seahorse: Seahorse Agilent XF HS Mini (v 3.0.0.41)<br>cryoEM EPU 1.12
Data analysis	Microscopy: FIJI (v1.5.2)<br>Statistics: GraphPad Prism (v8.4.3)<br>FACS: FlowJo (v10.8.1)<br>Proteomics: Perseus (v1.6.12.0)<br>Coot 0.96<br>Phenix 1.19<br>Chimera 1.14<br>ChimeraX 1.6.1<br>cryoSPARC4.6<br>RELION4

For manuscripts utilizing custom algorithms or software that are central to the research but not yet described in published literature, software must be made available to editors and reviewers. We strongly encourage code deposition in a community repository (e.g. GitHub). See the Nature Portfolio guidelines for submitting code & software for further information.

Data

Policy information about availability of data

All manuscripts must include a data availability statement. This statement should provide the following information, where applicable:

- Accession codes, unique identifiers, or web links for publicly available datasets
- A description of any restrictions on data availability
- For clinical datasets or third party data, please ensure that the statement adheres to our policy

The atomic coordinates were deposited in the Protein Data Bank (PDB) under accession numbers 9I4X (T. gondii complex III with ELQ 300/ATQ), 9I4Y (T. gondii complex IV), 9H8T (C. sabaeus CIII with ATQ), 9G9T (T. gondii CIII with ELQ-300). The cryo-EM maps have been deposited in the Electron Microscopy Data Bank (EMDB) under the respective accession numbers: EMD-52621 (T. gondii complex III with ELQ 300/ATQ), EMD-52622 (T. gondii complex IV), EMD-51939 (C. sabaeus CIII with ATQ), EMD-51157 (T. gondii CIII with ELQ-300). The atomic coordinates that were used in this study are: PDB 7O3C (murine III2-IV supercomplex), 5IY5 (cytochrome c) and 3CX5 (complex III with bound cyto-chrome c). Full versions of all gels are provided in the source file. All the data will be publicly available. Source data are provided with this paper. The mass spectrometry proteomics data have been deposited to the ProteomeXchange Consortium via the PRIDE partner repository with the dataset identifier PXD053932.

Research involving human participants, their data, or biological material

Policy information about studies with human participants or human data. See also policy information about sex, gender (identity/presentation), and sexual orientation and race, ethnicity and racism.

Reporting on sex and gender	N/A
Reporting on race, ethnicity, or other socially relevant groupings	N/A
Population characteristics	N/A
Recruitment	N/A
Ethics oversight	N/A

Note that full information on the approval of the study protocol must also be provided in the manuscript.

Field-specific reporting

Please select the one below that is the best fit for your research. If you are not sure, read the appropriate sections before making your selection.

☒ Life sciences ☐ Behavioural & social sciences ☐ Ecological, evolutionary & environmental sciences

For a reference copy of the document with all sections, see nature.com/documents/nr-reporting-summary-flat.pdf

Life sciences study design

All studies must disclose on these points even when the disclosure is negative.

Sample size	For cryo-EM analysis sample sizes were determined based on our earlier works and experience, sufficient to obtain reliable results, as confirmed by the estimated resolution.<br>All other experiments were repeated multiple times independently as indicated. Sample sizes are consistent with previously published studies for each technique. e.g:<br>JC-1: n=8 (Mallo et al., 2021; PMID: 34523684)<br>Seahorse: n=6 (Silva et al., 2023; PMID: 38079448)<br>Competition assay: n=4 (Mühleip et al., 2021; PMID: 33402698)<br>Immunoprecipitation: n=4 (Silva et al., 2023; PMID: 38079448)<br>Replication assay: n=4 (Maclean et al., 2024; PMID: 39207139)<br>TEM: n=100 (Mühleip et al., 2021; PMID: 33402698)<br>MitoSox: n=4 (Aghabi et al., 2023; PMID: 37339985)
Data exclusions	For cryo-EM structure determination, particles that were not respiratory supercomplex were discarded by classification.
Replication	All experiments were repeated multiple times independently as indicated in the text. Experiments on parasites in Figure 3 H came from 6 independent experiments and ED6M,O came from 4 independent biological replicates, where each experiment was set up (parasite culture initiated and measurements taken) independently on different days. For the flow cytometry competition assay, the same cultures were measured by flow cytometry after each passage, for 6 passages, to determine the proportion of red and green fluorescent parasites. 4 independent experiments were undertaken. Experiments on parasites in Figure 3G came from independent experiments (parental and ApiCox10-KO JC1 n=8; parental and ApiCox10-KO JC1 and valinomycin, n=8). For all experiments all replicates were successful and variability that exists between replicates was reported.

	For TEM analysis in Extended data figure J-L, 100 mitochondrial profiles were identified per strain and cristae number and mitochondria area calculated. Mitochondrial profiles came from numerous parasites (individual parasites contain 1-5 mitochondrial profiles), and from 2 separate TEM preparations.
Randomization	Cryo-EM map resolution estimates by Fourier Shell Correclation were performed using half-maps from random half-sets.
Blinding	Investigators were not blinded during data acquisition or analysis in line with standard practice.

Reporting for specific materials, systems and methods

We require information from authors about some types of materials, experimental systems and methods used in many studies. Here, indicate whether each material, system or method listed is relevant to your study. If you are not sure if a list item applies to your research, read the appropriate section before selecting a response.

Materials & experimental systems

n/a	Involved in the study
☐	☒ Antibodies
☐	☒ Eukaryotic cell lines
☒	☐ Palaeontology and archaeology
☒	☐ Animals and other organisms
☒	☐ Clinical data
☒	☐ Dual use research of concern
☒	☐ Plants

Methods

n/a	Involved in the study
☒	☐ ChIP-seq
☐	☒ Flow cytometry
☒	☐ MRI-based neuroimaging

Antibodies

Antibodies used	Primary antibodies:<br>Rat Anti-HA (Roche, clone 3F10, ROAHAHA)<br>Mouse Anti-FLAG (ThermoFisher Scientific, clone FG4R, MA1-91878)<br>Rabbit anti-TOM40<br>Rabbit anti-MYS<br>Rabbit anti-GAP45<br>Mouse anti-TY<br><br>Secondary antibodies:<br>Anti-mouse IRDye 800CW (Li-COR 926-32210)<br>Anti-rabbit IRDye 680RD (Li-COR 926-68071)<br>Anti-Rat IRDye 800CW (Li-COR 926-32219)<br>Anti-Rat IgG H&L HRP (abcam, 6845)<br>Anti-Rabbit IgG H&L HRP (Promega, W401B)<br>Alexa Fluor Goat anti-Rabbit 594 (Invitrogen, A-11012)<br>Alexa Fluor Goat anti-Mouse 488 (Invitrogen, A-11001)
Validation	Commercial antibodies were validated by the manufacturer as described on manufacturer's website. Specificity in Toxoplasma was tested by immunoblot, running the eptiope tagged line along with a parental line. For example anti-HA in Fig ED6C and anti-FLAG in Fig. ED9C.<br>Non-commercial antibodies were validated in the following publications:<br>Mouse anti-Ty (Bastin et al 1996)<br>Rabbit anti-GAP45 (Plattner et al., 2008)<br>Rabbit anti-MYS (Ovciarikova et al., 2017)<br>Rabbit anti-TOM40 (van Dooren et al., 2016)

Eukaryotic cell lines

Policy information about cell lines and Sex and Gender in Research

Cell line source(s)	HFF-1 (ATCC SCRC-1041)<br>RHTATiΔku80 Sheiner et al (2011) "A systematic screen to discover and analyze apicoplast proteins identifies a conserved and essential protein import factor"<br>VERO (ATCC CCL-81)
Authentication	HFF cells were authenticated by ATCC using intraspecific STR analysis. Vero cells were not authenticated by STR analysis. Toxoplasma lines were validated by PCR and sequencing where appropriate.
Mycoplasma contamination	Testing for mycoplasma was not performed.

Commonly misidentified lines
(See ICLAC register)

No commonly misidentified lines were used.

Plants

Seed stocks

Report on the source of all seed stocks or other plant material used. If applicable, state the seed stock centre and catalogue number. If plant specimens were collected from the field, describe the collection location, date and sampling procedures.

Novel plant genotypes

Describe the methods by which all novel plant genotypes were produced. This includes those generated by transgenic approaches, gene editing, chemical/radiation-based mutagenesis and hybridization. For transgenic lines, describe the transformation method, the number of independent lines analyzed and the generation upon which experiments were performed. For gene-edited lines, describe the editor used, the endogenous sequence targeted for editing, the targeting guide RNA sequence (if applicable) and how the editor was applied.

Authentication

Describe any authentication procedures for each seed stock used or novel genotype generated. Describe any experiments used to assess the effect of a mutation and, where applicable, how potential secondary effects (e.g. second site T-DNA insertions, mosiacism, off-target gene editing) were examined.

Flow Cytometry

Plots

Confirm that:

☒ The axis labels state the marker and fluorochrome used (e.g. CD4-FITC).

☒ The axis scales are clearly visible. Include numbers along axes only for bottom left plot of group (a 'group' is an analysis of identical markers).

☒ All plots are contour plots with outliers or pseudocolor plots.

☒ A numerical value for number of cells or percentage (with statistics) is provided.

Methodology

Sample preparation

Parasites of the indicated strain were filtered through a 3-micron polycarbonate filter, treated and stained as indicated in the methods, before centrifugation at 1500 x g and resuspension in FACS buffer (25 mM HEPES, 5 mM EDTA, 1% v/v FBS).

Instrument

BD FACSCelesta (BD Biosciences).

Software

Data were acquired using BD FACSDiva v9 and analysed using the FlowJo v10.8.1 software (BD biosciences).

Cell population abundance

The only whole-cells present in the population were Toxoplasma tachyzoites. These were seperated from host cell debris using the gating strategy described below.

Gating strategy

Populations were gated on FSC-A and SSC-A to gate on the parasite population and separate it from host cell debris. Single cells were then selected (FSC-A vs FSC-H) for downstream analysis of fluorescence.

☒ Tick this box to confirm that a figure exemplifying the gating strategy is provided in the Supplementary Information.
