# Supplementary figures and images for: Structure, assembly and inhibition of the Toxoplasma gondii respiratory chain supercomplex
Source: Nat Struct Mol Biol. 2025 May 19;32(8):1424–33. doi: 10.1038/s41594-025-01531-7 (PMC12350165; doi:10.1038/s41594-025-01531-7)

Figure 3: source data

Panel A

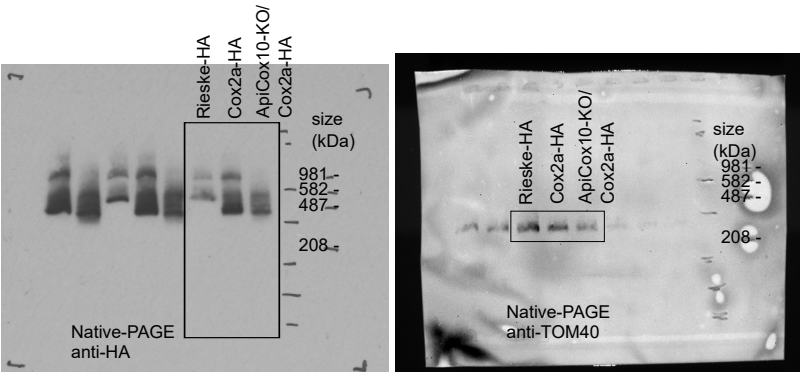

Panel B

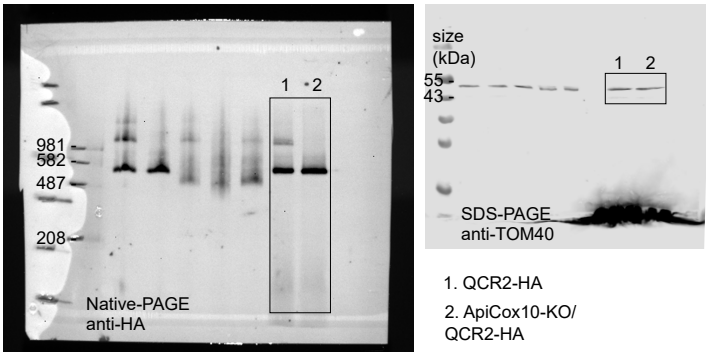

Panel C

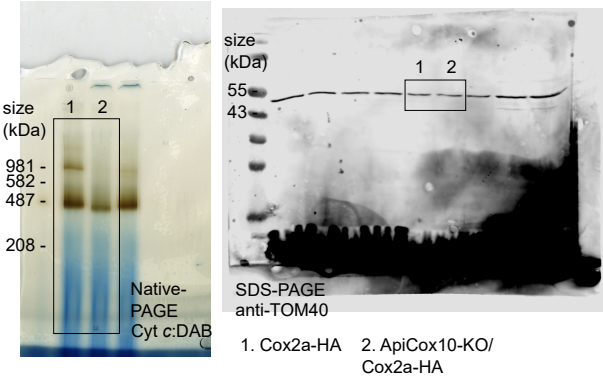

Panel D

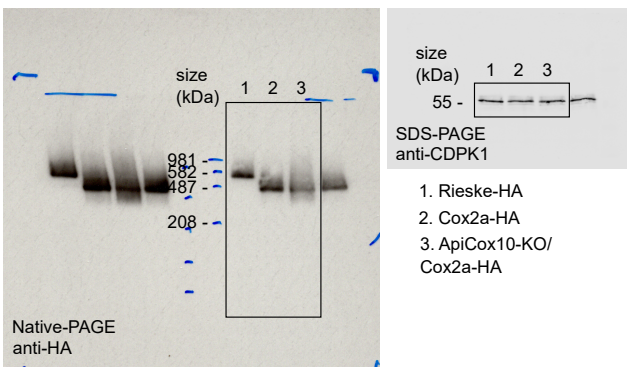

Panel E

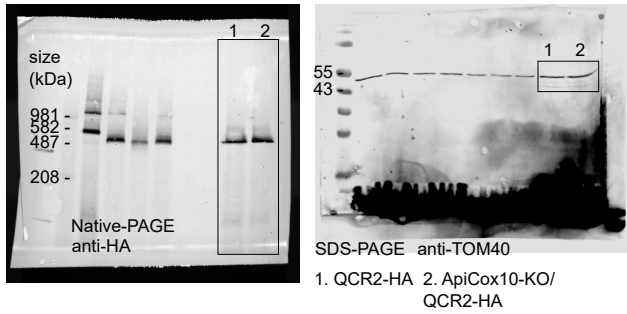

Panel F

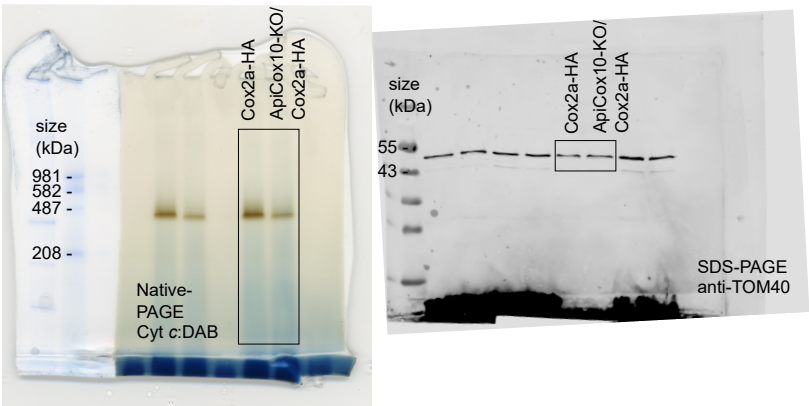

Supplement: Supplementary file 7 — Uncropped gel images (pdf) and original data values (xlsx). [file 41594_2025_1531_MOESM7_ESM.zip › Source_Data_Figure_3.pdf]

Extended data 6: source data

Panel B

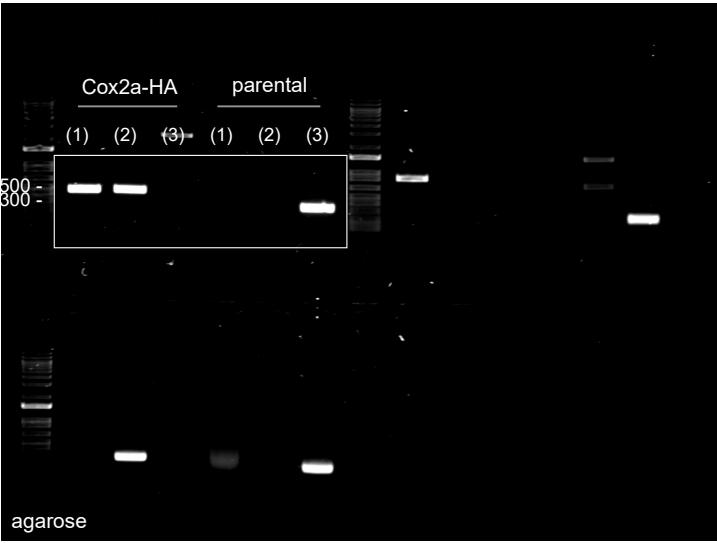

Panel C

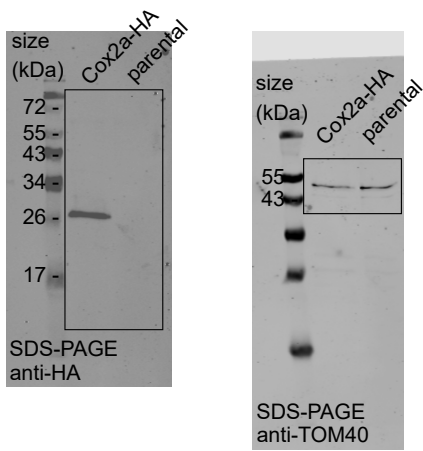

Panel E

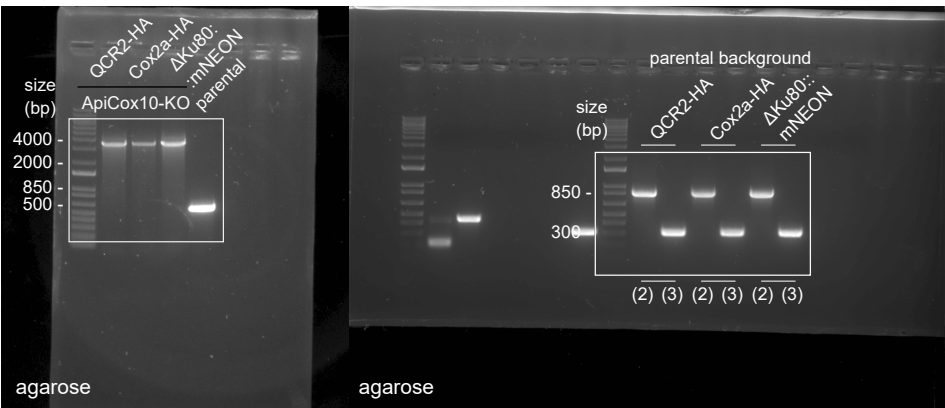

Panel F

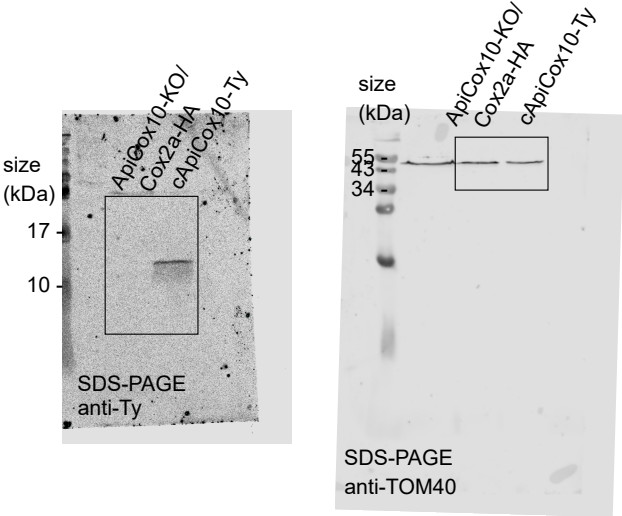

Panel I

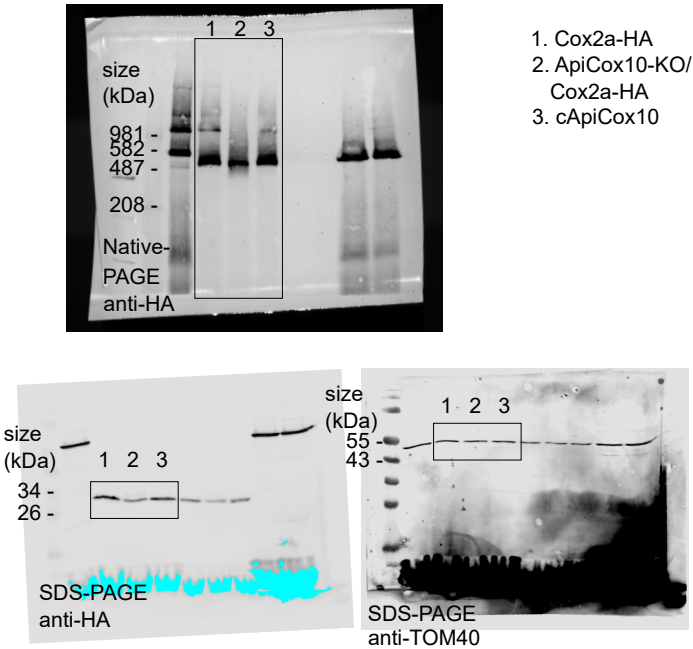

Supplement: Supplementary file 10 — Raw blots and gels (pdf) and raw images and data values (xlsx). [file 41594_2025_1531_MOESM10_ESM.zip › Source_Data_ED6.pdf]

Extended data 9: source data

Panel B

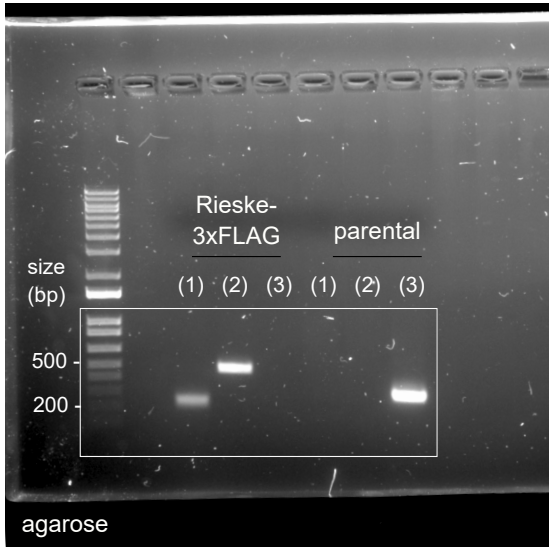

Panel C

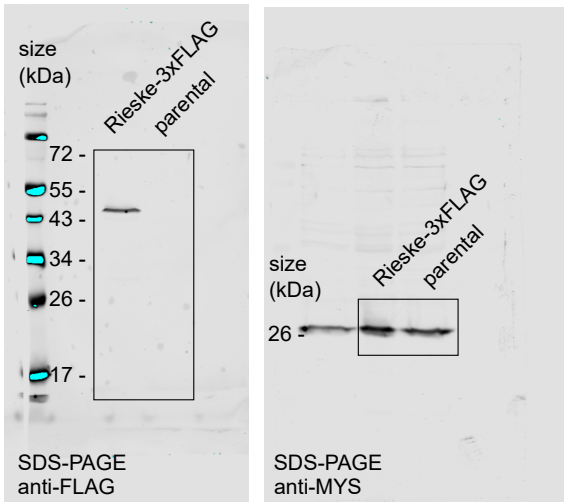

Panel D

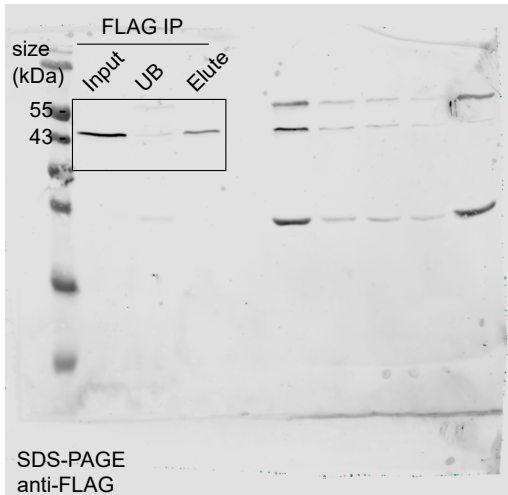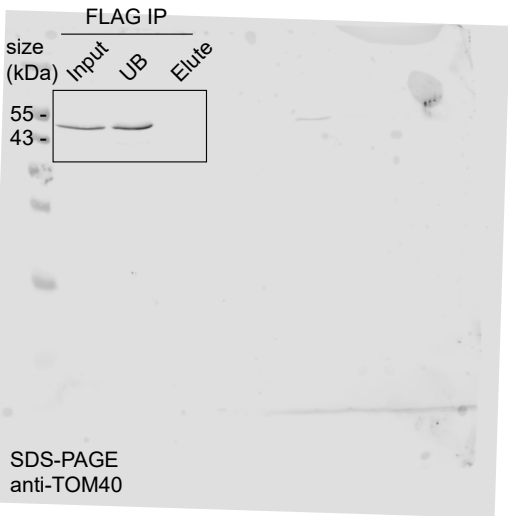

Panel E

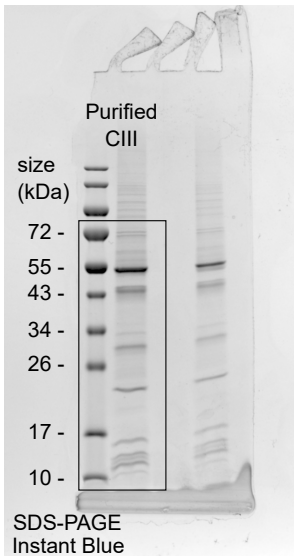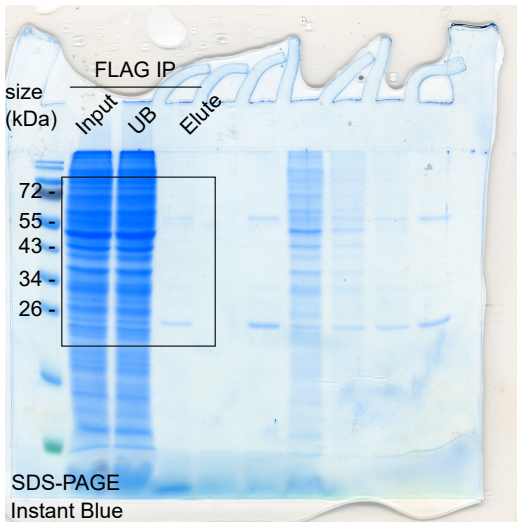

Supplement: Supplementary file 11 — Uncropped blots and gels. [file 41594_2025_1531_MOESM11_ESM.pdf]
